# Characterizations of a neutralizing antibody broadly reactive to multiple gluten peptide:HLA-DQ2.5 complexes in the context of celiac disease

**DOI:** 10.1038/s41467-023-44083-4

**Published:** 2023-12-22

**Authors:** Yuu Okura, Yuri Ikawa-Teranishi, Akihiko Mizoroki, Noriyuki Takahashi, Takashi Tsushima, Machiko Irie, Zulkarnain Harfuddin, Momoko Miura-Okuda, Shunsuke Ito, Genki Nakamura, Hiroaki Takesue, Yui Ozono, Masamichi Nishihara, Kenta Yamada, Siok Wan Gan, Akira Hayasaka, Shinya Ishii, Tetsuya Wakabayashi, Masaru Muraoka, Nishiki Nagaya, Hiroshi Hino, Takayuki Nemoto, Taichi Kuramochi, Takuya Torizawa, Hideaki Shimada, Takehisa Kitazawa, Makoto Okazaki, Junichi Nezu, Ludvig M. Sollid, Tomoyuki Igawa

**Affiliations:** 1 Translational Research Division, Chugai Pharmaceutical Co., Ltd., Tokyo, Japan; 2 Research Division, Chugai Pharmaceutical Co., Ltd., Kanagawa, Japan; 3 Chugai Pharmabody Research Pte. Ltd., Singapore, Singapore; 4 Translational Research Division, Chugai Pharmaceutical Co., Ltd., Kanagawa, Japan; 5 R&D Portfolio Management Department, Chugai Pharmaceutical Co., Ltd., Tokyo, Japan; 6 Department of Immunology, Oslo University Hospital, Oslo, Norway; 7 Institute of Clinical Medicine, University of Oslo, Oslo, Norway

**Keywords:** Antibody therapy, Autoimmunity, Gastrointestinal diseases, Molecular engineering

## Abstract

In human celiac disease (CeD) HLA-DQ2.5 presents gluten peptides to antigen-specific CD4^+^ T cells, thereby instigating immune activation and enteropathy. Targeting HLA-DQ2.5 with neutralizing antibody for treating CeD may be plausible, yet using pan-HLA-DQ antibody risks affecting systemic immunity, while targeting selected gluten peptide:HLA-DQ2.5 complex (pHLA-DQ2.5) may be insufficient. Here we generate a TCR-like, neutralizing antibody (DONQ52) that broadly recognizes more than twenty-five distinct gluten pHLA-DQ2.5 through rabbit immunization with multi-epitope gluten pHLA-DQ2.5 and multidimensional optimization. Structural analyses show that the proline-rich and glutamine-rich motif of gluten epitopes critical for pathogenesis is flexibly recognized by multiple tyrosine residues present in the antibody paratope, implicating the mechanisms for the broad reactivity. In HLA-DQ2.5 transgenic mice, DONQ52 demonstrates favorable pharmacokinetics with high subcutaneous bioavailability, and blocks immunity to gluten while not affecting systemic immunity. Our results thus provide a rationale for clinical testing of DONQ52 in CeD.

## Abstract (web summary)

Targeting gluten antigens presents a plausible therapy option for celiac disease. Here the authors generate and characterize a broadly neutralizing antibody recognizing more than 25 gluten peptide:HLA-DQ2.5 complexes, with structural analyses implicating its mode of interaction, and with mouse in vivo studies supporting its therapeutic feasibility.

## Introduction

Celiac disease (CeD) is an autoimmune disorder where the ingestion of gluten drives intestinal damage^1^. The patients have gluten-specific CD4^+^ T cells that recognize gluten in the context of disease-associated human leukocyte antigen (HLA) molecules, specifically HLA-DQ2.5, which is carried by the majority of patients. The global prevalence of biopsy-confirmed CeD is 0.7%^2^. To date, there is no approved drug to treat CeD. The only available treatment is a life-long gluten-free diet (GFD). While this treatment is well established, adherence to GFD is a burden that lowers their quality of life^3^. Even under a strict diet, 20–50% of diagnosed patients suffer from persistent symptoms, mainly due to unintentional gluten intake^4,5^. Thus, a new treatment that will resolve persistent symptoms and replace GFD could improve the lives of CeD patients.

Given the central role of gluten-specific T cells in CeD pathogenesis^1,2^, a therapeutic avenue in CeD could be to block the presentation of gluten peptides to CD4^+^ T cells by HLA-DQ2.5 with a neutralizing antibody. However, this approach is hindered by pharmacokinetic and safety issues. Because HLA plays a central role in physiological immunity, the systemic blockade of HLA-DQ2.5 may increase the risk of infection^6^ and affect thymic T cell education^7^. In addition, due to abundant expression of HLA-DQ2.5 systemically^8^, antibody targeting HLA-DQ2.5 would be very rapidly cleared from the body due to target-mediated clearance. This would require an unrealistically large antibody dosage and frequent intravenous injections as a life-long treatment.

A possible approach to overcome this challenge is to generate an antibody that binds to HLA-DQ2.5 in complex with selected gluten peptides and thereby inhibits the interaction with T cell receptors (TCRs). The TCR-like antibody is such an antibody, combining the favorable properties of a monoclonal antibody (for example, good pharmacokinetics, stability, and manufacturability) with the ability to specifically target peptide:HLA complexes (pHLA) like TCRs with strong affinity. TCR-like antibodies have been reported against HLA-DQ2.5:DQ2.5-glia-α1a^9^ or HLA-DQ2.5:DQ2.5-glia-α2^10^. These TCR-like antibodies are highly specific for a particular immunodominant gluten pHLA-DQ2.5. However, more than 80 % of HLA-DQ2.5+ CeD patients are responsive to multiple gluten epitopes other than DQ2.5-glia-α1a and DQ2.5-glia-α2^11–13^. In HLA-DQ2.5+ CeD, there are five distinct immunodominant epitopes found in wheat (DQ2.5-glia-α1a, DQ2.5-glia-α2, DQ2.5-glia-ω1, DQ2.5-glia-ω2) or barley (DQ2.5-hor-3a)^11^. In addition, more than thirty gluten epitopes with different amino acid sequences are known or predicted to be presented on HLA-DQ2.5, and conceivably the T cell response to all these epitopes drive pathology in CeD^14^. Therefore, unlike TCR-like antibodies specific against such pathogenic gluten pHLA-DQ2.5, a TCR-like antibody that broadly neutralizes multiple distinct pathogenic gluten pHLA-DQ2.5 molecules, including those that have five immunodominant epitopes, would be a safe and effective therapy for the majority of HLA-DQ2.5+ CeD patients. However, the generation of such a TCR-like antibody is challenging due to low sequence homology of pathogenic gluten epitopes. Even among the five immunodominant epitopes, the sequence homology is 33–78 %^14^. In addition, the majority of TCRs are reported to discriminate between two highly homologous epitopes by distinguishing minor differences in pHLA-DQ2.5 topologies^15^. This further increases the difficulty of generating TCR-like antibodies that are broadly reactive to multiple distinct gluten pHLA-DQ2.5. To date, no broadly cross-reactive antibody against multiple pathogenic pHLA II has been reported.

In this study, we generate a broadly reactive, high affinity TCR-like antibody in bi-specific format, called DONQ52. It binds to more than twenty-five distinct gluten pHLA-DQ2.5, including all immunodominant epitopes, and blocks gluten dependent immune activation in vitro and in vivo, while preserving systemic immunity. A deeper structural analysis indicates that broad reactivity of DONQ52 is attributed to its flexible recognition of unique motif of gluten epitopes. Therefore, our study suggests that DONQ52 would potentially be clinically beneficial for HLA-DQ2.5+ CeD patients, thus providing a significant step toward the development of therapeutic interventions for CeD.

## Results

### Generation of TCR-like antibody broadly reactive to multiple distinct gluten pHLA-DQ2.5

**Fig. 1:**
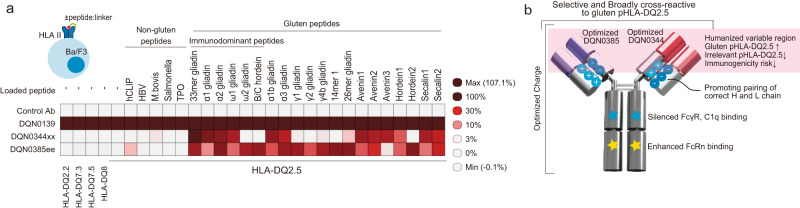
Key characteristics of lead antibodies and schematic drawing of DONQ52. **a** Lead antibodies binding to Ba/F3 cells expressing various pHLA II as determined by flow cytometry. Gating strategy to determine the MFI is shown in Supplementary Fig. 11a. Data are from an assay performed in triplicates (*n* = 3). Heat map represents relative mean MFI (%) of antibodies (DQN0344xx and DQN0385ee: 0.5 μg/mL, Control Ab: 10 μg/mL) to DQN0139 (anti-HLA-DQ neutralizing antibody, 10 μg/mL). **b** Schematic drawing of DONQ52.

To generate TCR-like antibodies broadly reactive to gluten pHLA-DQ2.5, we first immunized NZW rabbits with recombinant HLA-DQ2.5:33mer gliadin peptide (Supplementary Table 1). We used recombinant HLA-DQ2.5:33mer gliadin peptide as an immunogen to enrich cross-reactive antibody during affinity maturation process, since the 33mer gliadin peptide contains multiple pathogenic epitopes, such as DQ2.5-glia-α1a, DQ2.5-glia-α2 and DQ2.5-glia-α1b^16^. B cells from immunized rabbits were subjected to fluorescence-activated single-cell sorting to select B cells which bind to HLA-DQ2.5:33mer gliadin. To further screen for HLA-DQ2.5:33mer gliadin binder, more than 40,000 antibodies from B cell supernatants were screened for binding to various peptide:HLA II (pHLA II). Recombinant chimeric antibodies were then produced and screened to select antibodies broadly cross-reactive to gluten pHLA-DQ2.5 using flow cytometry with Ba/F3 cell panels expressing a variety of pHLA-DQ2.5 (Supplementary Table 2) or HLA II. DQN0344xx and DQN0385ee were identified as the lead antibodies, having demonstrated selectivity and cross-reactivity to multiple distinct gluten pHLA-DQ2.5 (Fig. 1a). The complementary binding specificity of the two antibodies prompted us to engineer them into a bi-specific IgG format consisting of a DQN0344xx arm and DQN0385ee arm to achieve extensive coverage against multiple gluten epitopes. The variable regions of the two lead antibodies were humanized, and then subjected to extensive protein engineering in line with previously reported methods^17,18^, to improve target binding cross-reactivity and affinities while maintaining selectivity, pharmacokinetics (PK), physicochemical properties, and immunogenicity. In this process, antibodies were mainly screened for binding to twenty-nine gluten pHLA-DQ2.5, including the five immunodominant epitopes as well as lack of binding to five pHLA-DQ2.5 that contain non-gluten HLA-DQ2.5 binding peptides (Supplementary Table 2). Variable regions of the lead antibodies were formatted into bi-specific immunoglobulin G (IgG) by engineering residues located in the interfaces of cognate heavy and light chains and two heavy chains to promote correct chain pairing. Furthermore, Fc of bi-specific IgG was engineered to enhance binding to neonatal Fc receptor at acidic pH to improve PK^19^, and to reduce binding to Fc gamma receptors and C1q to eliminate effector functions (Fig. 1b). Combined, these engineering techniques enabled us to generate DONQ52. As a control antibody, we generated DQN0139, which neutralizes HLA-DQ irrespective of the loaded peptides, and which has exact same Fc as DONQ52 constant region. Here we successfully generated TCR-like antibodies with broadly cross-reactive to gluten pHLA-DQ2.5 and enhanced their properties via multidimensional optimization.

### In vitro characterization of DONQ52

**Fig. 2:**
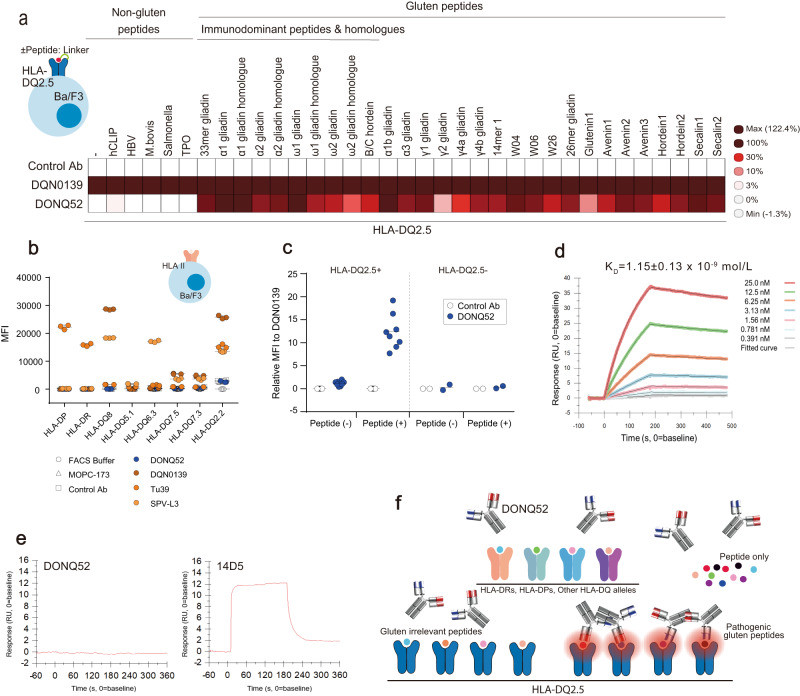
Binding characteristics of DONQ52. **a** Antibodies binding to Ba/F3 cells expressing various pHLA-DQ2.5 as determined by flow cytometry. Gating strategy to determine the MFI is shown in Supplementary Fig. 11a. Data are from an assay performed in triplicates (*n* = 3). Heat map represents relative mean MFI (%) of antibodies (DONQ52: 0.5 μg/mL, Control Ab: 10 μg/mL) to DQN0139 (10 μg/mL). Numerical data sets are shown in Supplementary Table 3. **b** Antibodies binding to non-HLA-DQ2.5, HLA II expressing Ba/F3 cell lines were determined by flow cytometry. Gating strategy to determine the MFI is shown in Supplementary Fig. 11a. Tu39 is anti-HLA-DR, DP, DQ antibody (mouse IgG2a). SPV-L3 is anti-pan HLA-DQ antibody (mouse IgG2a). MOPC-173 is mouse IgG2a isotype antibody. Control Ab is anti-KLH antibody (clone: IC17-SG181) as an isotype control for DONQ52 and DQN0139. Each binding antibody is colored as indicated. Data are from an assay performed in triplicates (*n* = 3) and are shown as mean ± SD. Numerical data sets are shown in Supplementary Table 4. **c** Antibodies binding to HLA-DQ2.5+ (*n* = 8) or HLA-DQ2.5- (*n* = 4) human B cells determined by flow cytometry. Relative values of MFI (%) of antibodies (DONQ52 (blue), Control Ab (gray): 10 μg/m) to DQN0139 (10 μg/mL) are shown. Peptide (-) means B cells cultured without the 33mer gliadin peptide, and Peptide (+) means B cells cultured with the 33mer gliadin peptide. Gating strategy to determine the MFI is shown in Supplementary Fig. 11b. Individual numerical data is shown in Supplementary Table 5. **d** Representative SPR sensorgram of the DONQ52 for binding to HLA-DQ2.5:33mer gliadin. *K*_*D*_ value was derived by 1:1 binding model. **e** Representative SPR sensorgram of the DONQ52 binding to the 33mer gliadin peptide itself, or 14D5, which is an anti-gliadin antibody (Abcam PLC). **f** Schematic representation of DONQ52 binding properties.

DONQ52 was tested for its binding selectivity using a panel of Ba/F3 cell lines expressing a variety of pHLA-DQ2.5 (Supplementary Table 2) or HLA II. DONQ52 was broadly reactive to pathogenic gluten pHLA-DQ2.5, while demonstrating no substantial binding to all tested gluten-irrelevant pHLA-DQ2.5 molecules, such as human CLIP pHLA-DQ2.5, hepatitis B virus (HBV) pHLA-DQ2.5, salmonella pHLA-DQ2.5, mycobacterium bovis pHLA-DQ2.5, and thyroid peroxidase (TPO) pHLA-DQ2.5 (Fig. 2a and Supplementary Table 3). These peptides were reported to bind to HLA-DQ2.5^20–23^. In addition, DONQ52 did not bind to other HLA II molecules (Fig. 2b and Supplementary Table 4). Because primary B cells express a plethora of different pHLA II (i.e., various endogenous pHLA II)^20,21^, we tested the binding of DONQ52 to primary human B cells highly expressing HLA-DQ, and confirmed that DONQ52 bound to HLA-DQ2.5 + B cells only in the presence of the 33mer gliadin peptides (Fig. 2c, Supplementary Table 5). The lack of staining of HLA-DQ2.5 + B cells in the absence of 33mer gliadin peptide suggests that DONQ52 has negligible off-target binding to irrelevant pHLA-DQ2.5. Surface plasmon resonance (SPR) experiments further confirmed that DONQ52 specifically bound to the HLA-DQ2.5:33mer gliadin with *K*_*D*_ = 1.15 ± 0.13 × 10^−9^ M (Fig. 2d), and not to the peptide itself. (Fig. 2e). Collectively, these findings demonstrate that DONQ52 recognizes HLA-DQ2.5 in the form of a complex with gluten peptides, and simultaneously possesses extensive cross-reactivity to various gluten epitopes yet with limited binding to irrelevant peptides. It suggests that DONQ52 would potentially block gluten dependent immunity while preserving systemic immunity. A schematic representation of the binding properties of DONQ52 is shown in Fig. 2f.

### In vitro broad neutralization of DONQ52

**Fig. 3:**
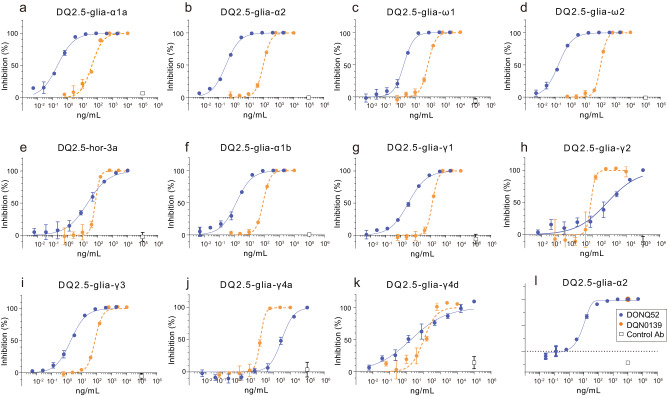
In vitro neutralization activity of DONQ52. In vitro neutralizing activity of DONQ52 (blue), positive control antibody (DQN0139) (orange), and negative control antibody (Control Ab, anti-keyhole limpet hemocyanin (KLH) antibody) (open square) on NFAT-Luc luciferase activity using αβTCR-knockout Jurkat-NFAT-Luc2 cells. Neutralizing activity against (**a**) DQ2.5-glia-α1a epitope, (**b**) DQ2.5-glia-α2 epitope, (**c**) DQ2.5-glia-ω1 epitope, (**d**) DQ2.5-glia-ω2 epitope, (**e**) DQ2.5-hor-3a epitope, (**f**) DQ2.5-glia-α1b epitope, (**g**) DQ2.5-glia-γ1 epitope, (**h**) DQ2.5-glia-γ2 epitope, (**i**) DQ2.5-glia-γ3 epitope, (**j**) DQ2.5-glia-γ4a epitope, (**k**) DQ2.5-glia-γ4d epitope are shown. Peptides used for stimulation and their concentrations are shown in Supplementary Table 6. Inhibitory effect of antibodies on TCR activation (%) of each well was calculated when taking a mean cps (count per second, luciferase activity) of the well in the absence of antigen peptide and antibody as 100 %, and a mean cps of the well in the presence of antigen without antibody as 0 %. Data are from an assay performed in triplicates (*n* = 3) and are plotted as mean ± SD. **l** In vitro neutralizing activity of DONQ52 (blue), DQN0139 (orange), and Control Ab (open square) on IL-2 production using human CD4^+^ T cell expressing TCRs specific for DQ2.5-glia-α2. Inhibitory effect of antibodies on IL-2 production (%) of each well was calculated when taking a mean IL-2 concentration of the well in the absence of 33mer gliadin peptide at 1 μM and antibody as 100%, and a mean IL-2 concentration of the well in the presence of antigen without antibody as 0%. Data are from the assay performed in triplicates (*n* = 3) and are plotted as mean ± SD.

To determine the in vitro neutralizing potency of DONQ52, endogenous αβTCR-knockout Jurkat-NFAT-Luc2 cells were generated by clustered regularly interspaced short palindromic repeat (CRISPR)- caspase 9 (Cas9) system. It was further engineered to express one of the following HLA-DQ2.5 restricted TCRs specific for all five immunodominant epitopes^11^ (DQ2.5-glia-α1a, DQ2.5-glia-α2, DQ2.5-glia-ω1, DQ2.5-glia-ω2 and DQ2.5-hor-3a), or 6 additional CeD relevant epitopes (DQ2.5-glia-α1a, DQ2.5-glia-γ1, DQ2.5-glia-γ2, DQ2.5-glia-γ3, DQ2.5-glia-γ4a, DQ2.5-glia-γ4d) (Supplementary Table 6). Each of these cells was co-cultured with the HLA-DQ2.5 + B lymphoblastoid cell line (IHW09023, The International Histocompatibility Working Group (IHW)) and the corresponding gluten peptides at non-saturated TCR activation-inducing concentrations (Supplementary Fig. 1, Supplementary Tables 1 and 6). TCR activation was detected by luciferase activity. As a result, DONQ52 demonstrated dose-dependent neutralization against all eleven gluten epitopes tested (Fig. 3a–k), and its median inhibitory concentrations (IC_50_) against the five immunodominant epitopes were between 0.162 and 27.9 ng/ml (Supplementary Table 6), which were notably stronger than that of DQN0139 (Fig. 3a–k). Because significant interleukin-2 (IL-2) release has been confirmed following gluten challenges in CeD patients with well-controlled GFD^24^, IL-2 is one of the most reliable CeD relevant CD4^+^ T cell activation markers. To determine whether DONQ52 blocks IL-2 production from human CD4^+^ T cells expressing TCRs specific for HLA-DQ2.5-presenting pathogenic gluten epitopes, we isolated human CD4^+^ T cells from healthy donors and transduced TCRs specific for DQ2.5-glia-α2. These cells were co-cultured with IHW09023 and the 33mer gliadin peptide at 1 μM which is submaximal IL-2-inducing concentrations (Supplementary Fig. 2). As a result, DONQ52 dose-dependently inhibited IL-2 production from human CD4^+^ T cell expressing TCRs specific for DQ2.5-glia-α2 (Fig. 3l). Taken together, DONQ52 neutralized all tested gluten epitopes including all immunodominant epitopes, suggesting that DONQ52 can neutralize multiple gluten epitopes in HLA-DQ2.5+ CeD patients.

### Structural basis to assess further cross-reactivity of DONQ52

**Fig. 4:**
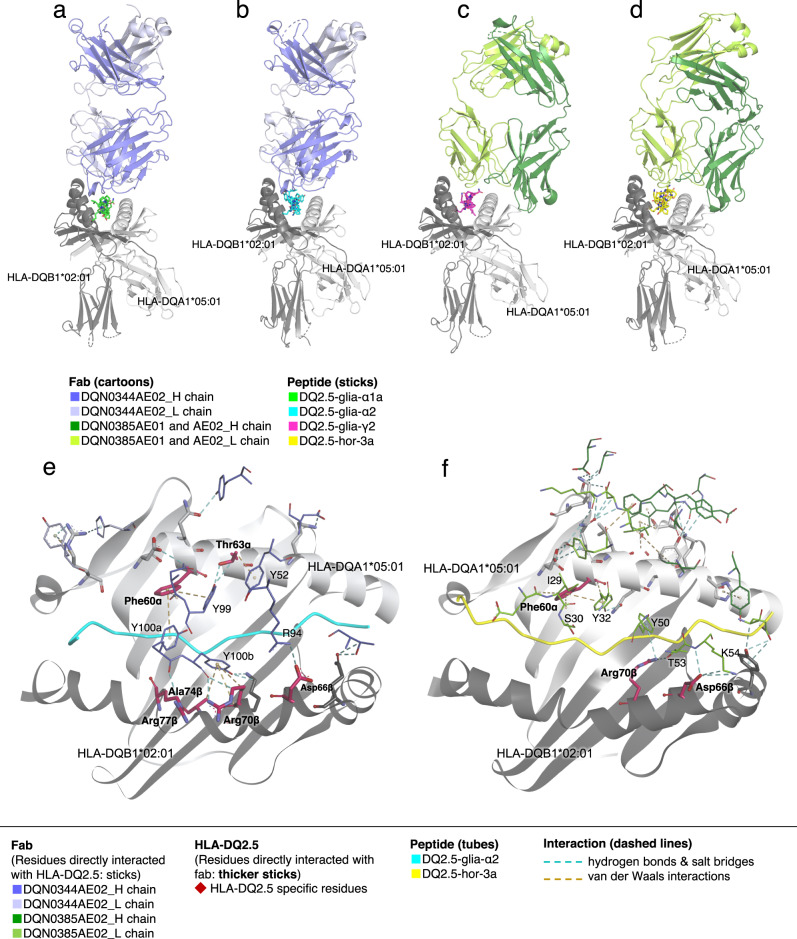
Structural basis of Fab recognition of gluten pHLA-DQ2.5. **a** Structures of Fab DQN0344AE02 and HLA-DQ2.5:DQ2.5-glia-α1a, (**b**) Fab DQN0344AE02 and HLA-DQ2.5:DQ2.5-glia-α2, (**c**) Fab DQN0385AE01 and HLA-DQ2.5:DQ2.5-glia-γ2, and (**d**) Fab DQN0385AE02 and HLA-DQ2.5:DQ2.5-hor-3a. Fab colors, and peptide are colored as indicated. **e** Interactions of the Fab DQN0344AE02 with HLA-DQ2.5 α and β chains. **f** Interactions of Fab DQN0385AE02 with HLA-DQ2.5 α and β chains. Fab residues directly interacting with HLA-DQ2.5 allotype specific residues are represented as sticks with single-letter codes, and HLA-DQ2.5 allotype specific residues directly interacting with Fab are represented as thicker sticks with bold three-letter codes. Cyan colored dotted lines represent hydrogen bonds and salt bridges, and other colored dotted lines represent van der Waals interaction.

We confirmed that DONQ52 was broadly reactive to multiple distinct gluten pHLA-DQ2.5 in several ways. However, CeD patients have individually variable responsiveness to multiple gluten epitopes including gluten epitopes which have yet to be evaluated or identified^11–14^. To estimate clinical efficacy of DONQ52 against HLA-DQ2.5+ CeD patients with individually distinctive pathogenic gluten epitopes, we addressed how each arm of DONQ52 structurally recognizes multiple gluten epitopes with different amino acid sequences by analyzing the X-ray crystal structure of the complexes between fragment antigen-binding region (Fab) of the antibodies and antigen. We determined the structures of DQN0344AE02 - HLA-DQ2.5:DQ2.5-glia-α1a, DQN0344AE02 - HLA-DQ2.5:DQ2.5-glia-α2, DQN0385AE01 - HLA-DQ2.5:DQ2.5-glia-γ2, and DQN0385AE02 - HLA-DQ2.5:DQ2.5-hor-3a at 2.8 Å, 2.1 Å, 2.8 Å, and 2.2 Å resolutions, respectively (Supplementary Table 7). According to the electron density map (Supplementary Fig. 3), the regions of epitope/paratope interactions are clearly visible, suggesting that the structures are suitable for analyzing and discussing the interactions between the Fabs and pHLA-DQ2.5. Notably, the antibodies used in this study are engineered variants obtained in the process of optimization of humanized antibodies, yet the identity to DONQ52 is high (VH > 93%, VL > 94%), and the paratope residues are conserved between DQN0344AE02/DQN0385AE01 and DONQ52, and except for Glu28, between DQN0385AE02 and DONQ52. Thus, while some caution should be applied in transferring interpretations of these structures to an antibody that has altered Fc region and bivalent Fab regions, these structures still provide valuable insights into the molecular basis for the reactivity of DONQ52. Our Fabs bound to all gluten pHLA-DQ2.5 in a conventional TCR-like manner^25^ (Fig. 4a–d). We contrasted the interactions of our Fabs with interactions observed in HLA-DQ2.5:DQ2.5-glia-α2 specific TRAV26-1/TRBV7-2+ TCRs (S16, JR5.1, D2)^26^, or a TCR-like antibody specific to HLA-DQ2.5:DQ2.5-glia-α2 (3.C11)^10^. To understand the structural mechanism underlying the broad specificity of DONQ52, we first analyzed buried surface areas (BSAs) and electrostatic surface potentials (ESPs) of the structures. The BSAs of our Fabs and pHLA complexes range between 1875 Å^2^ and 2661 Å^2^, similar to the BSAs of the previously reported complexes (Supplementary Fig. 4). The electrostatic charge of S16 TCR and 3.C11 is asymmetrically distributed along the peptide (Supplementary Fig. 5a, b), whereas the charge of DQN0344 is asymmetrically distributed vertically across the peptide (Supplementary Fig. 5c, d). DQN0385 has an asymmetric charge distribution along the peptide, similar to S16 TCR and 3.C11, yet the polarity of the surface potential is opposite (Supplementary Fig. 5a, b, e, f). Neither BSAs nor ESPs provided obvious clues as to why our Fabs demonstrate broad specificity to pHLA-DQ2.5.

Analysis of interacting residues in the Fabs does however provide insight into their recognition of HLA-DQ2.5, and also as to why they have broad reactivity to gluten epitopes. First, like the HLA-DQ2.5:DQ2.5-glia-α2 specific TCRs^26^ or TCR-like antibody^10^, both DQN0344 and DQN0385 form interaction with Asp66β and Arg70β. These residues are unique to HLA-DQ2.5, indicating that these interactions play critical role in the specific recognition of the HLA-DQ2.5 allotype. Further, for DQN0344, three consecutive Tyr residues (Tyr99, Tyr100a and Tyr100b) in the CDR3-VH loop contact Phe60α, Thr63α, Arg70β, Ala74β, and Arg77β of HLA-DQ2.5 (Fig. 4e). In addition, Arg94 in FR3-VH and Tyr52 in the CDR2-VH loop contact Asp66β and Thr63β of HLA-DQ2.5, respectively (Fig. 4e). In DQN0385, Ile29, Ser30, and Tyr32 in the CDR1-VL loop contact Phe60α of HLA-DQ2.5, and Tyr50, Thr53, and Lys54 in the CDR2-VL loop contact Asp66β and Arg70β of HLA-DQ2.5 (Fig. 4f).

**Fig. 5:**
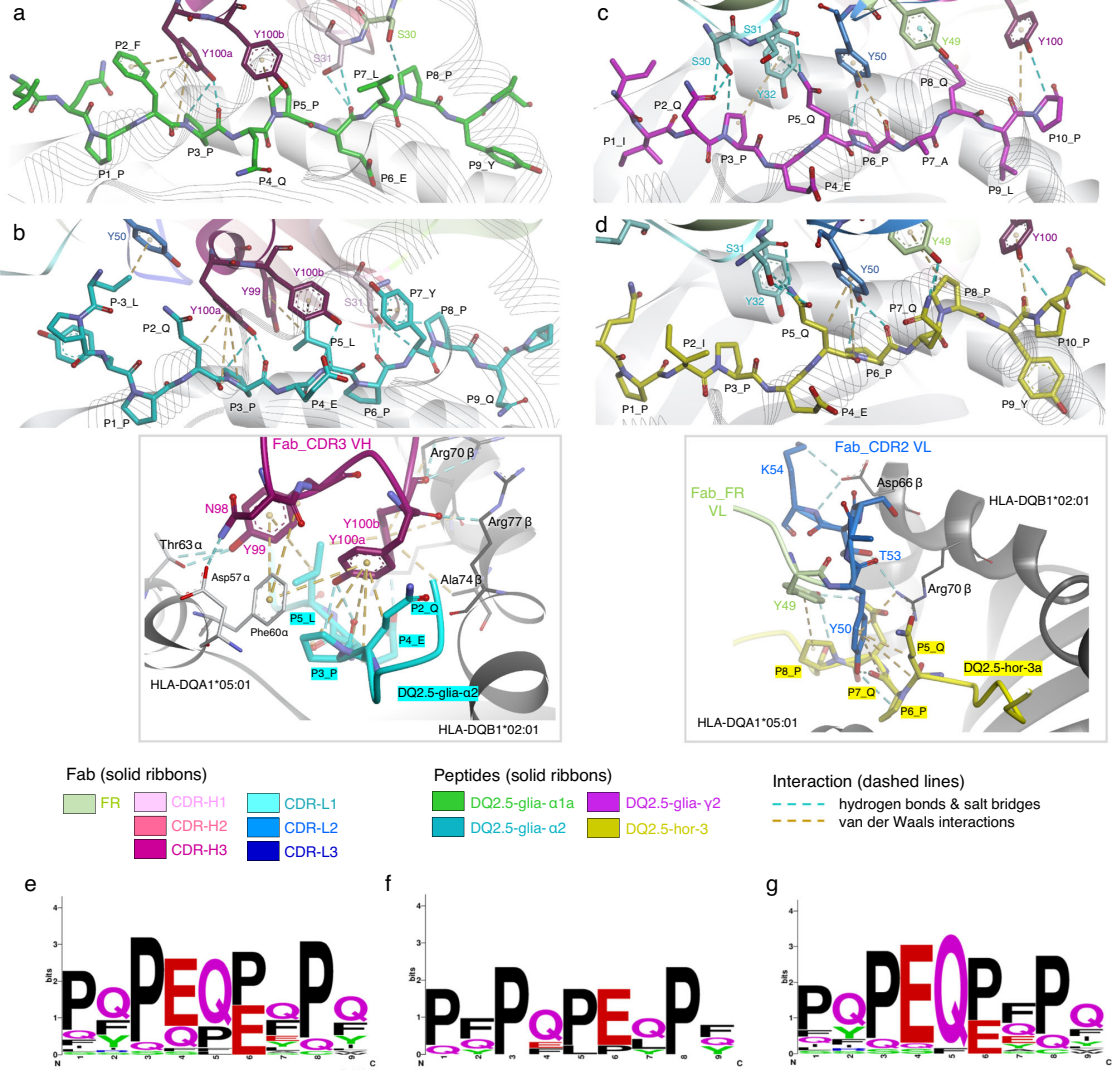
Interaction of Fabs with HLA-DQ2.5 loaded gluten peptides. **a** Interactions of DQN0344AE02 with HLA-DQ2.5:DQ2.5-glia-α1a. Fab and peptides residues are represented as sticks with single-letter codes. Cyan colored dotted lines represent hydrogen bonds and salt bridges, and ocher colored dotted lines represent van der Waals interaction. CDRs, FR, and peptide are colored as indicated. **b** Interactions of DQN0344AE02 with HLA-DQ2.5:DQ2.5-glia-α2. Box insert: Key DQN0344 interactions to P3, P5 of DQ2.5-glia-α2 and surrounding HLA-DQ2.5 residues. HLA-DQ2.5 allotype specific residues directly interacting with Fab are represented as sticks with three-letter codes. **c** Interactions of DQN0385AE01 with HLA-DQ2.5:DQ2.5-glia-γ2. **d** Interactions of DQN0385AE02 with HLA-DQ2.5:DQ2.5-hor-3a. Box insert: Key DQN0385 interactions to P5-P8 of DQ2.5-hor-3a and surrounding HLA-DQ2.5 specific residues. **e** Sequence motif of all HLA-DQ2.5 restricted pathogenic gluten core 9-mer epitopes are taken from ref. ^14^. **f** Sequence motif of those HLA-DQ2.5 restricted pathogenic gluten core 9-mer epitopes that have non-bulky residues (Pro or Leu) at P5. **g** Sequence motif of those HLA-DQ2.5 restricted pathogenic gluten core 9-mer epitopes that have bulky residues (Gln or Phe) at P5. Sequence logos are generated by WebLOGO.

Second, the structural basis for the broad recognition of gluten epitopes by DQN0344 and DQN0385 have striking commonalities. In DQN0344, being reactive with both HLA-DQ2.5:DQ2.5-glia-α1a (PFPQPELPY) and HLA-DQ2.5:DQ2.5-glia-α2 (PQPELPYPQ), Tyr100a in CDR3-VH contacts the side-chain and backbone of P3-Pro in both gluten epitopes by arching down and deeply penetrating into the peptide binding cleft (Figs. 4e, 5a, b). Tyr100b and Tyr99 surrounding Tyr100a in CDR3-VH play auxiliary roles (Figs. 4e, 5a, b). These Tyr residues contact the side-chain of P5-Pro/Leu in the two gluten epitopes (Fig. 5a, b). In addition, Ser31 in the CDR1-VH interacts with a backbone of P6-Glu in DQ2.5-glia-α1a or a backbone of P6-Pro and a side chain of P7-Tyr of DQ2.5-glia-α2 (Fig. 5a, b). As shown in Fig. 1a, DQN0344 tends not to bind to gluten pHLA-DQ2.5 when there is a bulky residue (i.e., Gln or Phe) at P5 of the peptide. This lack of reactivity is due to the steric hindrance between arch-forming Tyr residues (Tyr99, Tyr100a, Tyr100b) and bulky residues at P5 of gluten epitopes. Of note, in gluten T cell epitopes the P3 residue appears to always be Pro when there is a non-bulky residue (i.e., Pro or Leu) at P5 (Fig. 5e, f)^14^, indicating that DQN0344 is cross-reactive to all gluten epitopes carrying this feature.

In the case of DQN0385, which is reactive to both HLA-DQ2.5:DQ2.5-glia-γ2 (IQPEQPAQL) and HLA-DQ2.5:DQ2.5-hor-3 (PIPEQPQPY), Tyr50 in CDR2-VL contacts the side-chain and backbone of P6-Pro in both gluten epitopes by arching down into the peptide binding cleft (Fig. 5c, d). Similarly for both epitopes, Tyr32 in CDR1-VL contacts the side-chain of P5-Gln (Fig. 5c, d). Tyr49 in FR2-VL contacts P8-Pro or P8-Gln, supporting the binding (Fig. 5c, d). Generally, pathogenic gluten epitopes present P6-Pro and/or P8-Pro which facilitate the specific deamidation of P4-Gln and/or P6-Gln, the critical anchor positions within the pathogenic gluten epitopes^27^. Since P5 is mostly Gln when it is not Pro nor Leu (Fig. 5e, g), the cross-reactivity of DQN0385 to gluten epitopes with P5-Gln complements DQN0344, which recognizes P5-Pro and P5-Leu. As an exception, HLA-DQ2.5:DQ2.5-glia-γ1 has Phe at P5; however, this is strongly recognized by DQN0385 (Fig. 1a). Superposing of the DQ2.5-glia-γ1 peptide structure (Supplementary Fig. 6) from PDB ID: 5KSA on the DQN0385AE01 - HLA-DQ2.5:DQ2.5-glia-γ2 structure reveals that both Tyr32 and Tyr50, which interact with P5-Gln and P6-Pro in other peptides, could coordinately interact with P5-Phe in DQ2.5-glia-γ1.

In both DQN0344 and DQN0385, multiple Tyr residues form a series of protuberance structures, and depending on the bulkiness of epitope residues, those multiple Tyr residues flexibly and effectively interacts with diverse gluten epitopes (Supplementary Fig. 7). Such a series of protuberance structures are not observed in S16 TCR or 3.C11. In DQN0385 as compared with DQN0344, the series of protuberance structures are less pronounced. The Tyr residues are spread along the conformation of gluten epitopes (Supplementary Fig. 7), giving a shallower penetration of the peptide binding cleft (Fig. 5d). This explains why DQN0385 tolerates bulkiness of epitope residues.

Taken together, multiple Tyr residues, especially Tyr100a (Tyr100b, Tyr99) in DQN0344 CDR3-VH and Tyr-50 (Tyr32) in DQN0385 CDR2-VL (CDR1-VL) appear to have critical roles in the broad binding to various gluten pHLA-DQ2.5. Unlike the previously reported TCRs or antibody, the use of multiple flexible Tyr residues for their recognition of unique Pro/Gln residues in gluten epitopes leads to broad cross-reactivity to pathogenic gluten pHLA-DQ2.5. DONQ52, being bi-specific representing the reactivity of both DQN0344 and DQN0385, likely would cover most CeD-relevant T cell gluten epitopes.

### Single dose pharmacokinetic study of DONQ52 in HLA-DQ2.5 transgenic mice

**Fig. 6:**
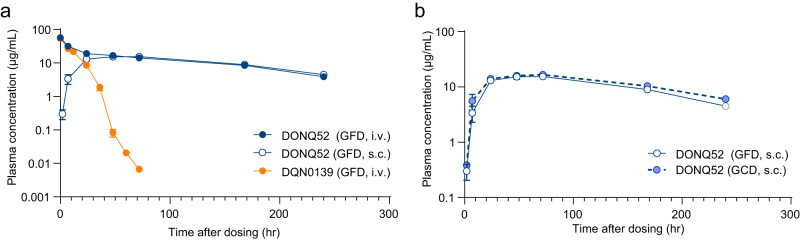
Single dose pharmacokinetic profile of antibodies in DQ2.5 Tgm. Antibody plasma levels were measured by enzyme-linked immunosorbent assay (ELISA). **a** Time course of the plasma concentration of antibodies in DQ2.5 Tgm fed with GFD after a 2 mg/kg intravenous (DQN0139 (orange) and DONQ52 (blue)) or subcutaneous (DONQ52) injection (open circle). **b** Time course of the plasma concentration of DONQ52 in DQ2.5 Tgm fed with GFD (solid line) or GCD (dotted line) after a 2 mg/kg intravenous or subcutaneous injection. Data was analyzed using the concentrations of DONQ52 in plasma excluding anti-drug antibody-positive samples. Data are from sparse sampling of *n* = 8 mice/group and *n* = 4 mice/sampling point, plotted as mean ± SD.

In single dose PK analysis of DQN0139 and DONQ52 in endogenous major histocompatibility complex (MHC) II knockout and HLA-DQ2.5 transgenic mice (DQ2.5 Tgm)^28^, DQN0139 exhibited extremely rapid clearance and short T_1/2_ (0.276 day at 2 mg/kg intravenously) (Fig. 6a and Table 1); this is because it binds to all endogenously expressed HLA-DQ2.5, which accelerates target-mediated clearance. On the other hand, DONQ52 demonstrated a long T_1/2_ (4.09 day at 2 mg/kg intravenously) (Fig. 6a and Table 1) consistent with human IgG PK in mice^29^, since it recognizes HLA-DQ2.5 presenting gluten epitopes. The subcutaneous bioavailability of DONQ52 was 80.5% at 2 mg/kg (Table 1), denoting high subcutaneous bioavailability. Moreover, gluten-containing diet (GCD) did not affect the pharmacokinetic profile of DONQ52 (Fig. 6b and Table 1), suggesting that ingestion of gluten would only lead to a negligible amount of HLA-DQ2.5 being complexed with gluten peptide. Altogether, DONQ52, unlike DQN0139, had a long half-life and high subcutaneous bioavailability without affecting gluten ingestion. It suggests that DONQ52 would be a durable therapeutic option for CeD.

**Table 1:** Pharmacokinetic parameters of DONQ52 and DQN0139 in DQ2.5 Tgm

PK Parameters^a^	DONQ52: 2 mg/kg intravenously	DQN0139: 2 mg/kg intravenously	DONQ52: 2 mg/kg subcutaneously
Feed	GFD	GFD	GFD
T_1/2_ (day)	4.09	0.276	2.66
AUCinf (mg × d/mL)	149	26.1	120
CL (mL/d/kg)	13.5	76.5	N/A^b^
CL/F (mL/d/kg)	N/A^b^	N/A^b^	16.6
F (%)	N/A^b^	N/A^b^	80.5

Time-points for T_1/2_ calculation were automatically set by WinNonlin based on the result of the individual time profile in plasma concentrations. For calculation of the pharmacokinetics parameters, values below the limit of quantitation (BLQ) were treated as 0 μg/mL.

^a^Calculated parameters are T1/2 (terminal phase half-life), AUCinf (area under the curve to infinity), CL (clearance), CL/F (subcutaneous clearance) and F (bioavailability). F was calculated by the following formula: F (%) = (AUCinf / dose) at subcutaneous dosing/(AUCinf/dose) at intravenous dosing × 100.

^b^N/A, not applicable.

### In vivo inhibition by DONQ52 of antigen dependent immune response in HLA-DQ2.5 transgenic mice

**Fig. 7:**
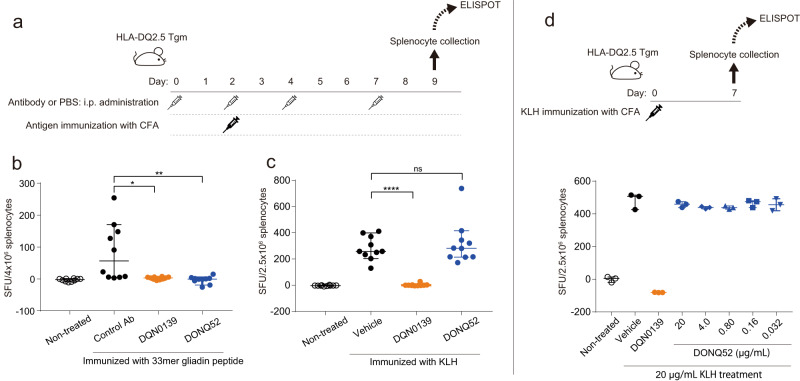
In vivo and ex vivo neutralizing activity of DONQ52 in DQ2.5 Tgm. **a** Outline of experiments and (**b**, **c**) results. IL-2 ELISPOT assay with splenocyte of DQ2.5 Tgm immunized with the (**b**) 33mer gliadin peptide, or (**c**) the KLH. **b** DONQ52 (*n* = 10, blue), DQN0139 (*n* = 10, orange) or Control Ab (*n* = 10, black) were intraperitoneally administered to DQ2.5 Tgm three times a week from day 0, followed by the 33mer gliadin peptide immunization at day 2. Mice in a non-treated group (*n* = 10, open circle) received no antibody. Splenocytes isolated at day 9 were subjected to IL-2 ELISPOT assay stimulated with the 33mer gliadin peptide. Results are in spot forming cells (SFC)/4 × 10^6^ splenocytes. **c** DONQ52 (*n* = 10, blue), DQN0139 (*n* = 10, orange) or vehicle (PBS) (*n* = 10, black) were intraperitoneally administered to DQ2.5 Tgm three times a week from day 0, followed by the KLH immunization at day 2. Mice in a non-treated group (*n* = 10, open circle) received no antibody. Splenocytes isolated at day 9 were subjected to IL-2 ELISPOT assay stimulated with KLH. Results are in SFC/2.5 × 10^6^ splenocytes. Points: mean spot counts of each animal (triplicate wells), bars: median spot counts of each group, error bars: 95% CI (±1.96 SD), ns: not significant (*p* = 0.6576), *p = 0.0031, **p = 0.0017, and ***p < 0.0001: these denote statistical significance for the differences compared to the control group, as determined by a two-way ANOVA and Dunnett’s multiple comparison test. **d** Outline of experiments and result. DQ2.5 Tgm were subcutaneously immunized with KLH at day 0. Seven days after immunization, splenocytes were isolated and subjected to mouse IL-2 ELISPOT assay. Splenocytes (2.5 × 10^6^ cells/well) were cultured with the KLH (20 μg/mL) and DONQ52 (blue), DQN0139 (orange), or vehicle (black), or without the KLH (open circle) on ELISPOT plates. Anti-MHC I antibody (50 μg/mL) was added to all wells to block MHC I mediated CD8 + T-cell activation. Results are in SFC/2.5 × 10^6^ splenocytes. Data are plotted from an assay performed in triplicates; bars: median spot counts of each group; error bars: 95 % CI (±1.96 SD).

To evaluate the efficacy of DONQ52 in vivo, we used DQ2.5 Tgm. The 33mer gliadin peptide (Fig. 7a) or the keyhole limpet hemocyanin (KLH) (Fig. 7b) immunization induced antigen reactive T cells in vivo. While the intraperitoneal administration of DQN0139 prevented T cell induction by both antigens, DONQ52 specifically blocked the 33mer gliadin peptide-dependent T cell induction (Fig. 7a, b). Furthermore, DONQ52 effectively blocked DQ2.5-glia-γ2 epitope-dependent T cell induction at a stable plasma concentration of around 30 μg/mL, a concentration which is readily achievable in humans^30,31^ (Supplementary Fig. 8). Although the final proof of effect will come from the testing of drug PK and efficacy in the clinical trials with CeD patients, this result suggests that even epitopes that are poorly neutralized by DONQ52 in vitro (Fig. 3, Supplementary Table 6) will be potently blocked by DONQ52 in vivo at clinically realistic plasma antibody levels. During the experiment, DONQ52 administration did not cause any abnormalities in the general conditions of the mice. In addition, ex vivo activation of the KLH-dependent T cells from the KLH immunized mice was completely blocked by DQN0139 at 20 μg/mL, but not by DONQ52 at the same concentration (Fig. 7c). Taken together, DONQ52 administered in vivo specifically blocked the 33mer gliadin peptide-dependent immune response, while not affecting a response to KLH.

## Discussion

We reported DONQ52, a novel TCR-like antibody in bi-specific format which broadly neutralizes more than twenty-five distinct gluten pHLA-DQ2.5, offering an effective, safe, and patient-friendly therapeutic for CeD.

Although potential therapeutic TCR-like antibodies specific for HLA-DQ2.5:DQ2.5-glia-α1a^9^ or HLA-DQ2.5:DQ2.5-glia-α2^10^ have been previously reported, their targets are limited to these two immunodominant epitopes. As there exists multiple pathogenic gluten epitopes^11–14^, and as the majority of HLA-DQ2.5+ CeD patients have T cells being responsive to multiple gluten epitopes other than the DQ2.5-glia-α1a and DQ2.5-glia-α2 epitopes^11–13^, blocking of only a limited number of gluten pHLA-DQ2.5 molecules would not suffice for therapy. Because of the presence of TCRs cross-reactive to some pathogenic gluten or non-gluten pHLA-DQ2.5^15,32,33^, TCR-like antibodies need to be cross-reactive to multiple pathogenic pHLA-DQ2.5 to be therapeutically effective. The advantages of DONQ52 over TCR-like antibodies specific for certain gluten epitopes in targeting HLA-DQ2.5 are summarized (Supplementary Fig. 9).

While therapeutic TCR-like antibodies have been previously reported, their target has been mostly limited to HLA I:tumor antigen^34^. Since TCR-like antibodies recognize both HLA and loaded peptides, unlike antibodies targeting conventional antigen, various TCR-like antibodies to HLA I are reported to be cross-reactive to homologous peptides^35^. TCR-like antibodies targeting HLA I:tumor antigens are often engineered to eliminate cross-reactivity to homologous peptides in order to minimize off-target side effects^34,35^. In this study, we took advantage of the intrinsically cross-reactive nature of TCR-like antibodies and identified unprecedentedly broad cross-reactive antibodies against distinct gluten pHLA-DQ2.5 involved in CeD from immunized rabbits. Rather than eliminating cross-reactivity, we engineered the lead antibodies to have desired cross-reactivity and binding affinity to more than twenty-five gluten pHLA-DQ2.5 molecules while avoiding irrelevant pHLA-DQ2.5 binding. To date, exploiting the cross-reactive nature of TCR-like antibody for therapeutic use has not been previously reported.

For gluten epitopes to be pathogenic, they must contain unique Pro- and Gln-rich motifs. This is strongly suggested by the fact that [1] pathogenic gluten epitopes, being unusually rich in Pro, have a specific rigid conformation that lead different gliadin epitopes into a strictly similar conformation^36^ and which is resistant to degradation in the gastrointestinal tract^37^; [2] P6- and P8-Pro facilitate the deamidation of P4- and P6-Gln, resulting in a strong anchor, which produces the pathogenic form of gluten epitopes^27,38^; [3] P5-Gln, P6-Pro, and P8-Pro/Gln are crucial for CeD relevant T cell recognition^39,40^. Structural analysis demonstrated that paratopes of DONQ52 contain an unusually high number of Tyr residues and these multiple Tyr residues flexibly recognize those Pro- and Gln-rich motifs common to pathogenic gluten epitopes, regardless of the amino acids adjacent to each Pro and Gln. Complementary recognition of those Pro- and Gln-rich motifs by Tyr residues in the two DONQ52 arms explains the broad cross-reactivity to various pathogenic gluten pHLA-DQ2.5 molecules. These structural biology studies indicate that DONQ52 likely would recognize all pathogenic gluten epitopes, including those yet to be evaluated or identified. This suggests that DONQ52 could improve gluten dependent pathogenesis in HLA-DQ2.5+ CeD. In addition, we assume that such flexible recognition by Tyr comes from its versatility in forming various intermolecular contacts, owing to its ability to form nonpolar, hydrogen-bonding and cation-π interactions^41^, suggesting that Tyr-engineering could be an effective approach to identify cross-reactive high affinity antibodies.

DONQ52 directly blocked TCR signals from multiple distinct gluten-derived peptides both in vitro and in vivo. More importantly, DONQ52 inhibited the 33mer gliadin-dependent but not the KLH-dependent immune responses in vivo. Considering the nature of CeD, long-term safety is a primary concern, and the systematic blockade of HLA-DQ2.5, as with DQN0139, has a risk of increasing infection^6^ and affecting thymic T cell education^7^. Since CeD requires life-long treatment, the convenience of long-term usage is also very important. Because DONQ52 exhibited long half-life and high bioavailability after subcutaneous injection in DQ2.5 Tgm, DONQ52 is predicted to be administered to CeD patients subcutaneously at weekly or longer intervals. Due to abundant expression of HLA-DQ2.5 systemically^8^, DQN0139 was rapidly cleared in DQ2.5 Tgm, thus a DQN0139-like antibody would require large dosage and frequent intravenous infusion at a hospital. Moreover, since protein drugs targeting MHC class II are highly immunogenic^42^, long-term treatment with a DQN0139-like antibody would be hindered by the formation of anti-drug antibodies. The advantages of DONQ52 over anti-HLA-DQ2.5 antibodies in targeting HLA-DQ2.5 (DQN0139-like antibody) are summarized (Supplementary Fig. 9).

This study needs to be interpreted in the context of potential limitations. Although the results of our in vitro and in vivo studies suggest that there is little chance that off-target binding would induce immunodeficiency and lack of response to infectious agents, DONQ52 is unlikely to affect systemic immunity in CeD patients. However, this study has not yet demonstrated that DONQ52 does not interfere with the HLA-DQ2.5 mediated immune response to “all” human cell-derived endogenous peptides or pathogen-derived peptide immune responses. Therefore, in the ongoing Phase 1 trial and subsequent clinical trials, we will carefully evaluate the safety in humans.

In summary, we generated a novel anti-HLA-DQ2.5 antibody, DONQ52, which is broadly reactive to a large collection of gluten pHLA-DQ2.5 molecules. Structural analysis revealed that this selectivity and broad reactivity is achieved by flexible recognition of common pathogenic proline-rich motif of gluten epitopes by multiple tyrosine residues present in the antibody paratope. Although the direct and specific targeting of pHLA-TCR interaction, which is the initial step in the critical pathway of the immune activation process, is a promising approach to treat various autoimmune diseases without affecting systemic immunity, no drugs have directly targeted this pathway to date. Our results suggest that DONQ52 directly and specifically blocks the initial step in the immune reaction to gluten without compromising systemic immunity in HLA-DQ2.5+ CeD patients. Structural analysis and preclinical studies demonstrated in this report provides a rationale for clinical testing of DONQ52 in patients with CeD, and phase 1 clinical trial of DONQ52 is currently underway.

## Methods

### Animal studies

All animal studies were performed according ARRIVE (Animal Research: Reporting of In Vivo Experiments) guidelines^43^. All procedures associated with this study were reviewed and approved by the Institutional Animal Care and Use Committee (IACUC) in Chugai Pharmaceutical Co., Ltd. The test facility is fully accredited by the Association for Assessment and Accreditation of Laboratory Animal Care International (AAALAC), a non-profit organization that promotes the humane treatment of animals in science through voluntary accreditation and assessment programs (http://www.aaalac.org). Animal care and experiments were performed according to the animal husbandry policy of Chugai Pharmaceutical Co., Ltd.

### Peptides

Peptides as described in Supplementary Table 1 were synthesized at GenScript. Peptides were re-constituted in PBS and stored at −80 °C until usage.

### Expression construct

A series of antibody variants were generated by introducing mutations into expression vectors using a PCR-based method. The design of the pHLA expression constructs was based on previously developed methods to produce soluble HLA II molecules^44^. Amino acid sequences of HLA II α chain and β chain were obtained from the IPD-IMGT/HLA Database (https://www.ebi.ac.uk/ipd/imgt/hla/allele.html). Recombinant HLA-DQ2.5:33mer gliadin is comprised of the extracellular domains from HLA-DQA1*05:01(C47S) and HLA-DQB1*02:01. The amino acid sequence encoding HLA-DQA1*05:01(C47S) was followed by c-fos leucine zipper^45^ and Flag-tag while the amino acid sequence encoding 33mer gliadin peptide (LQLQPFPQPELPYPQPELPYPQPELPYPQPQPF) covalently linked to HLA-DQB1*02:01, was followed by c-jun leucine zipper^45^, biotinylation sequence^46^ and 8 × His-Tag on the C-terminus. Recombinant HLA-DQ8:gliadin is comprised of the extracellular domains of HLA-DQA1*03:01 and HLA-DQB1*03:02. Gliadin peptide (QQYPSGEGSFQPSQENPQ) was covalently linked to the N-terminus of HLA-DQB1. Recombinant HLA-DQ5.1:DBY (ATGSNCPPHIENFSDIDMGE) is comprised of the extracellular domains from HLA-DQA1*01:01(C11Y) and HLA-DQB1*05:01. Recombinant HLA-DQ2.2:hCLIP (KLPKPPKPVSKMRMATPLLMQALPMGALP) is comprised of the extracellular domains from HLA-DQA1*02:01 and HLA-DQB1*02:02. Recombinant HLA-DQ7.5:hCLIP is comprised of the extracellular domains from HLA-DQA1*05:05 (C47S) and HLA-DQB1*03:01. For crystallization, the DQ2.5-glia-α1a peptide (QLQPFPQPELPYP), DQ2.5-glia-γ2 peptide (IIQPEQPAQLP), and DQ2.5-hor-3a peptide (EPEQPIPEQPQPYPQQP) were designed to be linked to the N-terminus of HLA-DQB1*02:01. The HRV 3 C protease cleavage site was incorporated at the N-terminus of the Fos and Jun leucine zipper sequences. To express HLA-DQ2.5:DQ2.5-glia-α2 (LPYPQPELPYPQP), the Fc region of human IgG1 was fused instead of the Fos and Jun leucine zipper.

### Antibody generation

Four NZW rabbits (1 female rabbit and 3 male rabbits, purchased from InVivos Pte Ltd. (Singapore)) were separately housed (1 rabbit/cage) in a temperature- and humidity-controlled, and specific pathogen-free room fed with a water and food under a 12-h light/12-h dark cycle. Twelve- to sixteen-week-old NZW rabbits were immunized intradermally with recombinant HLA-DQ2.5:33mer gliadin (50–100 μg/dose/rabbit). This dose was repeated 4–5 times over a 2-month period. One week after the final immunization, rabbits were euthanized under isoflurane-induced anesthesia for all blood draws and spleen sampling. B cells from 4 immunized NZW rabbits were mixed and subjected to Alexa Fluor 488-labeled HLA-DQ2.5:33mer gliadin, and biotin-labeled HLA-DQ5.1:DBY, HLA-DQ8:gliadin staining and fluorescence-activated single-cell sorting to select B cells which bind to HLA-DQ2.5:33mer gliadin, and not to HLA-DQ5.1:DBY, HLA-DQ8:gliadin using a cell sorter (FACSAria III, BD Biosciences, Franklin Lakes, NJ). B cells were cultured, and its supernatants were collected as previously described^47^. To further screen for HLA-DQ2.5:33mer gliadin binders, single cloned B cell culture supernatants containing secreted antibodies were subjected to various pHLA II (HLA-DQ2.5:33mer gliadin, HLA-DQ5.1:DBY, HLA-DQ8:gliadin, DQ2.2:hCLIP and DQ7.5:hCLIP) binding. Recombinant chimeric antibodies were then produced and screened to select antibody broadly cross-reactive to gluten pHLA-DQ2.5 s using flow cytometry with Ba/F3 cell panels expressing a variety of pHLA (Supplementary Table 2).

### Antibody engineering

Two original antibodies, DQN0344xx and DQN0385ee were humanized, followed by extensive mutational optimization of the antibodies’ heavy and light chains; each amino acid residue except for cysteine in all complementarily determining regions (CDRs) was replaced with a different natural amino acid, on a one-by-one basis to screen the effective mutations following previously described protocols^17,18^. Each variant was screened based on its binding properties against representative antigens such as HLA-DQ2.5:33mer gliadin peptide using SPR and/or flow cytometry analysis. Effective mutations discovered through this screening were combined to improve the properties of the two original mAbs DQN0344xx and DQN0385ee. Such comprehensive mutagenesis and combination were repeated until DONQ52 was created.

### Expression of antibody and recombinant pHLA

A series of antibody and recombinant pHLA were expressed using a FreeStyle293 Expression system (ThermoFisher Scientific) or Expi293 Expression System (ThermoFisher Scientific). 10 μg/mL kifunensine was supplemented to express the proteins for crystallization. A stable DONQ52-expressing Chinese Hamster Ovary (CHO) cell line was produced by a standard method and was used for large scale production.

### Purification of antibody

Conditioned media expressing antibody was applied to a column packed with Protein A resin and eluted with acidic solution. Fractions containing antibody were collected and subsequently subjected to a gel filtration column to remove high molecular weight species, if necessary. Target bi-specific antibody was further purified by ion exchange chromatography and mix mode chromatography to remove process-related impurities.

### Purification of pHLA

Conditioned media expressing the recombinant proteins was incubated with an immobilized metal affinity chromatography resin, followed by elution with imidazole. Fractions containing the recombinant protein were collected and subsequently subjected to a gel filtration column to remove high molecular weight species. HLA-DQ2.5:DQ2.5-glia-α2 was purified by affinity chromatography using Protein A and subsequent gel filtration chromatography. The recombinant pHLA molecules were biotinylated in a site-specific manner using the enzyme BirA, if necessary.

### Establishment of HLA II expressing Ba/F3 cell lines

Ba/F3 cells were obtained from Riken Cell bank. Amino acid sequences of HLA II α chain protein and HLA II β chain protein were obtained from the IPD-IMGT/HLA Database (https://www.ebi.ac.uk/ipd/imgt/hla/allele.html). EcoRI restriction enzyme sequence was attached at the N-terminal of the insert followed by KOZAK sequence (GAATTCCACC) While stop codon replicates were attached at the C-terminal of the insert followed by NotI restriction enzyme sequence (GCGGCCGC). Signal sequence of DQB1*02:01 was determined by Genetyx. All designed amino acid sequences were converted into mouse codon optimized cDNA sequences and inserted into EcoRI/NotI cloning site in pCXND3 vector or pCXZD1 vector which is a zeocin-resistant variant of the pCXND3 vector^48^. Schematics of insert sequence strategies of HLA-DR, HLA-DP and HLA-DQ molecules, and schematics of insert sequence strategies of the complex formed by HLA-DQ2.5 and peptide are described in Supplementary Fig. 10. All corresponding peptide sequences are listed in Supplementary Table 2. Five μg of each plasmid was mixed with 5 μL of 10× NEBuffer 3.1 (New England Biolabs), 5 μL PvuI restriction enzyme (New England Biolabs), and 35 μL of Nuclease free water. Mixture is incubated at 37 °C for 2 h and plasmids were then linearized. Electroporation was conducted according to the protocol of SG Cell line 4D-nucleofector X-kit (LONZA). Each of the linearized HLA II α chain plasmids and corresponding HLA II β chain plasmids were simultaneously introduced into Ba/F3 cell line by electroporation (LONZA, 4D-Nucleofector X). Transfected cells were then cultured with complete media (RPMI1640, Thermo Fisher Scientific) containing 9.0 vol% FBS (Bovogen), 90 units/mL Penicillin-Streptomycin (Thermo Fisher Scientific), 0.90 ng/mL recombinant mouse IL-3 (R&D Systems) for 1 day, after which they were supplemented with Geneticin (Thermo Fisher Scientific) and Zeocin (Invivogen) with or without additional complete media to select and expand cells.

### Surface plasmon resonance

Binding of DONQ52 to recombinant HLA-DQ2.5:33mer gliadin or the 33mer gliadin peptide was assessed using the Biacore T200 instrument (GE healthcare) at 25 °C and pH 7.4. Experiments were performed in triplicates. For the binding to recombinant HLA-DQ2.5:33mer gliadin, varying concentrations of HLA-DQ2.5:33mer gliadin were injected over DONQ52 captured on a ProAG immobilized CM4 surface, followed by a dissociation phase. The kinetic binding parameters were determined using a 1:1 binding model in the Biacore T200 Evaluation Software Version 2.0 (GE Healthcare). For the binding to the 33mer gliadin, anti-gliadin antibody (clone 14D5, Abcam) was used as a control antibody. A concentration of 2000 nmol/L 33mer gliadin peptide was injected over DONQ52 or 14D5 captured on ProAG immobilized CM4 surface, followed by a dissociation phase. The obtained sensorgram was analyzed to evaluate the binding of DONQ52 to the 33mer gliadin peptide.

### Antibody binding to Ba/F3 cell lines expressing pHLA-DQ2.5

DONQ52 (0.5 μg/mL), control Ab (10 μg/mL, anti-KLH antibody, generated in house), or DQN0139 (10 μg/mL, anti-HLA-DQ neutralizing antibody, generated in house) was incubated with 1 × 10^5^ cells/100 μL/well Ba/F3 cell panels (Supplementary Table 2) in FACS buffer (DPBS(−) containing 2% heat inactivated FBS (Nichirei) and 2 mM EDTA (Thermo Fisher Scientific)) for 30 min at room temperature, followed by antibody detection with × 50 diluted Goat F(ab′)2 anti-Human IgG, Mouse ads-PE (Southern Biotech) using flow cytometry (BD LSRFortessa X-20, Becton, Dickinson and Company). Gating strategy to determine the MFI is shown in Supplementary Fig. 11. MFI was calculated using BD FACSDiva Ver.8.0.1 software (Becton, Dickinson and Company).

### Antibody binding to Ba/F3 cell lines expressing HLA II

Ten μg/mL of DONQ52, control Ab (anti-KLH antibody, clone IC17-SG181), DQN0139, SPV-L3 (anti-pan HLA-DQ antibody, Beckman Coulter), Tu39 (anti-HLA-DR, DP, DQ antibody, BioLegend), or MOPC-173 (mouse IgG2a isotype antibody, BioLegend) was incubated with 1 × 10^5^ cells/100 μL/well Ba/F3 cells expressing HLA II in FACS buffer for 30 min at room temperature, followed by antibody detection with ×50 diluted Goat F(ab′)2 anti-Human IgG, Mouse ads-PE (Southern Biotech), or Goat F(ab′)2 anti-Mouse IgG2a, Human ads-PE (Southern Biotech) by flow cytometry (BD LSRFortessa X-20, Becton, Dickinson and Company). Gating strategy to determine the MFI is shown in Supplementary Fig. 11. MFI was calculated using BD FACSDiva Ver.8.0.1 software (Becton, Dickinson and Company). Staining antibodies used in flow cytometry experiments with experimental dilutions are listed in Supplementary Table 8.

### Antibody binding to human B cells

Antibody binding to B cells included in HLA-DQ2.5 positive PBMCs (*n* = 8), and HLA-DQ2.5 negative PBMCs (*n* = 4) was assessed by flow cytometry. Cryopreserved human PBMCs genotyped for HLA-DQ (LONZA) were used (Supplementary Table 5). PBMCs were seeded on a 24-well plate at 1 × 10^6^ cells/mL/wells in CTS OpTmizer T Cell Expansion SFM (Thermo Fisher Scientific). PBMCs were further cultured in the presence or absence of 100 μg/mL 33mer gliadin peptide (GenScript) for 3 h in a humidified atmosphere of 5% CO_2_ at 37 °C. PBMCs were re-distributed on a 96-well plate at 1–2 × 10^6^ cells/mL/wells, followed by incubation with DONQ52 or DQN0139 or control Ab at 10 μg/mL for 1 h at 4 °C. DQN0139 at 10 μg/mL was used as a positive control, and control Ab (anti-KLH antibody) at 10 μg/mL was used as a negative control. Cells were stained with AF647 labeled YG55H-rabIgG/YG55L-rabk (generated in house; secondary antibody, recognizing modified IgG heavy chain constant region with delta-GK-amide that applied to DONQ52, DQN0139, control Ab) and PE-Cy7 Mouse Anti-Human CD3 (Clone SP34-2, BD Biosciences), Pacific Blue Mouse Anti-Human CD14 (Clone M5E2, BD Bioscience), FITC Mouse Anti-Human CD16 (Clone 3G8, BD Bioscience), Anti-human CD20 PE (Clone 2H7, BD Bioscience), and PerCP anti-human HLA-DR Antibody (Clone L243, BD Bioscience). Data were acquired with BD FACSCant II (Becton, Dickinson and Company). Gating strategy to determine the MFI is shown in Supplementary Fig. 11. MFI was calculated using BD FACSDiva Ver.8.0.1 software (Becton, Dickinson and Company). Staining antibodies used in flow cytometry experiments with experimental dilutions are listed in Supplementary Table 8.

### Preparation of TCR expressing endogenous αβTCR-knockout Jurkat-NFAT-Luc2

*TRAC*- and *TRBC*-knockout Jurkat cell line was established as described^49^ with the following specifications. CD3 high expressing NFAT-RE-Luc2 Jurkat (Promega) was isolated by FACS Aria (Becton, Dickinson and Company). Before transfection, Cas9 (Takara Bio USA) and sgRNAs (Supplementary Table 9) were incubated to form RNP complex. The RNP complex was introduced to CD3 high expressing NFAT-RE-Luc2 Jurkat by following the protocol of Cell Line Nucleofector Kit V (LONZA). RNP complex-introduced cells were single cell sorted by FACS Aria. *TRAC* and *TRBC* sequences were then amplified by PCR and analyzed by 3730xl DNA Analyzer (Applied Biosystems). The sequence was confirmed by visual inspection using Genetyx, and Sequencher ver4.8. (Gene Codes Corp). Established αβTCR-knockout Jurkat-NFAT-Luc2 was further engineered to express HLA-DQ2.5:gluten epitope restricted TCRs (Supplementary Table 6). Electroporation of each TCR vector into αβTCR-knockout Jurkat-NFAT-Luc2 was conducted according to the protocol of SE Cell Line 4D-Nucleofector X Kit L (LONZA) just before in vitro neutralization assay.

### In vitro neutralization assay

Assay medium RPMI1640 (Nacalai Tesque), 10% vol heat inactivated FBS (Nichirei), 100 U/mL Penicillin-Streptomycin (Thermo Fisher Scientific), 1 × MEM (Thermo Fisher Scientific), 1 mM sodium pyruvate (Thermo Fisher Scientific) was used for the dilution of cells, antibodies, and peptides. Fifty μL of the HLA-DQ2.5 + IHW09023 mixture (purchased from Fred Hutchinson Cancer Research Center, 8.0 × 10^4^ cells/well) and each gluten peptide were distributed into 96 well round-bottom plates. Twenty-five μL of serially diluted antibodies were then applied in triplicate, and 25 μL of TCR transfected αβTCR-knockout Jurkat-NFAT-Luc2 (2.0 × 10^4^ cells/well) were finally distributed and incubated at 37 °C, and 5% CO_2_ overnight. After overnight culture, 50 μL of cultured cells were harvested and re-distributed in OptiPlate-96. Fifty uL of Bio-Glo Luciferase Assay Reagent (Promega) was then added and incubated at room temperature for 10 min, and luminescence resulting from TCR signaling was measured with EnVision (PerkinElmer). The correspondence of TCR and stimulating peptides and their final concentrations determined in a peptide titration study is described in Supplementary Table 6. The IC50 value was defined as the concentration at which there was a 50% decrease in luciferase activity. IC50 was determined by JMP15.0.0 (SAS Institute Inc) using inverse estimation in a non-linear regression model.

### Human CD4^+^ T cell isolation and TCR transduction

Research protocols and all uses of human materials have been approved by the Chugai Ethical Committee at Chugai Pharmaceutical Co., Ltd. All recruited volunteers provided written informed consent prior to blood collection. This process involved a detailed discussion of the study, potential risks and benefits, and participant rights. Only after obtaining informed consent blood was collected from volunteers. In this study, sex, gender, and age have been anonymized since these are considered not affecting the outcome of the experiment. Following blood collection from healthy volunteer, PBMCs were purified from the blood obtained with Ficoll-Paque PLUS (Miltenyi Biotech). CD4^+^ T cells were isolated from PBMCs by using CD4^+^ T Cell Isolation Kit, human (Miltenyi Biotech). Isolated CD4^+^ T cells were expanded for 3 days in 100% humidified 5% CO_2_ incubator set at 37 °C with AIM V medium (Thermo Fisher Scientific) supplemented with recombinant human IL-2 (R&D Systems) and anti-CD3 antibody (BioLegend). *TCR* gene specific for DQ2.5-α2 glia (D2 TCR, Supplementary Table 6) was inserted into lentivirus vector (pCDH-CMV-MCS-T2A-copGFP, System Bioscience), followed by introducing into lentivirus and prepare lentivirus particle by using FuGENE HD Transfection Regent (Promega). Lentivirus particle was then transduced to expanded CD4^+^ T cells by using Vectofusin-1 (Miltenyi Biotech) for 5 days in 100% humidified 5% CO_2_ incubator set at 37 °C with AIM V medium supplemented with recombinant human IL-2 and anti-CD3 antibody. Cells were then washed and cultured with assay medium overnight in 100% humidified 5% CO_2_ incubator set at 37 °C. Usage of Lentivirus is approved by company’s Biosafety Committee.

### In vitro IL-2 secretion assay

AIM V medium was used for the dilution of cells, antibodies, and peptides. Fifty μL of the HLA-DQ2.5 + IHW09023 mixture (4.0 × 10^5^ cells/well) with anti-human MHC Class I antibody (Clone: W6/32, Bio X Cell, Inc), anti-HLA-DR antibody (Clone: L243, Bio X Cell, Inc), and an equal amount of 33mer gliadin peptides (final concentration: 1 μM) or AIM V medium control were distributed in 96 well round-bottom plates. Fifty μL of serially diluted antibodies were then applied in triplicate, and an equal amount of DQ2.5-α2 glia specific TCR-transduced human CD4^+^ T cells (1.0 × 10^5^ cells / well) were finally distributed. The mixture was incubated at 37 °C, at 5% CO_2_ overnight. After overnight culture, supernatants were collected to measure IL-2 concentration using a Human IL-2 Quantikine ELISA Kit (R&D Systems) by using VersaMax Microplate Reader (Molecular Devices, LLC) and SoftMax Pro 6.4 (Molecular Devices, LLC).

### Structural analysis

The pHLA proteins were treated with PreScission protease (Cytiva) and subjected to affinity chromatography to remove the leucine zipper or Fc fragment. Although the Fab fragments of DONQ52 prototype antibodies (DQN0344AE02, DQN0385AE01 and DQN0385AE02) were used for crystallization, differences between the prototype antibodies and DONQ52 did not affect the interpretation of results (Supplementary Table 7). The Fab fragments of DONQ52 prototype antibodies (DQN0344AE02, DQN0385AE01 and DQN0385AE02) were prepared by a conventional method of limited digestion with Endoproteinase Lys-C (Roche), followed by sequential purification using a protein A column to remove the Fc fragment, and a cation exchange column and a gel filtration column to further purify Fab fragments. Each of these Fab fragments was mixed with the corresponding treated pHLA protein (Supplementary Table 7) as described above, and subsequently subjected to a gel filtration column equilibrated with 20–25 mM HEPES (pH 7.1) and 100 mM NaCl. Each of the complexes was concentrated to 8–11 mg/mL by ultrafiltration, and crystallized using the sitting drop vapor diffusion method at 293–294 K. The crystals which grew under the conditions in Supplementary Table 7 were frozen in liquid nitrogen.

Diffraction images from each single crystal were collected under a 95–100 K nitrogen stream. X-ray sources and detectors in the measurements are shown in Supplementary Table 7. Data processing of each dataset was performed using autoPROC^50^ (Global Phasing), which utilized XDS^51^ and AIMLESS^52^ in the CCP4 software suite^53^ and STARANISO^54^. The structures were determined by molecular replacement with Phaser^55^ using an in-house hIgG1 Fab and published HLA-DQ2.5 crystal structure^56^ (PDB ID: 1S9V) as search models. The models were built and refined with the Coot^57^ and Buster^58^ (Global Phasing). The data collection and structure refinement statistics are shown in Supplementary Table 7. X-ray structures of DQN0344AE02 - HLA-DQ2.5:DQ2.5-glia-α1a, DQN0344AE02 - HLA-DQ2.5:DQ2.5-glia-α2, DQN0385AE01 - HLA-DQ2.5:DQ2.5-glia-γ2 and DQN0385AE02 - HLA-DQ2.5:DQ2.5-hor-3a have four, one, two, and two sets of complexes in each asymmetric unit, respectively. Since there were no significant differences in the interface structures of Fab and HLA among the molecules in each asymmetric unit, we discussed their structural characteristics by focusing on one of them. Figures 4e, f, and  5a–d were prepared with BIOVIA Discovery Studio 2020. Figure 4a–d, and Supplementary Figs. 4–7 were generated with PyMOL version 2.3 (Schrödinger, LLC.). The calculations of BSA were performed with BIOVIA Discovery Studio 2020. The structures were deposited in the RCSB Protein Data Bank (https://www.rcsb.org/) with PDB IDs: 8W83 (DQN0344AE02 - HLA-DQ2.5:DQ2.5-glia-α1a), 8W84 (DQN0344AE02 - HLA-DQ2.5:DQ2.5-glia-α2), 8W85 (DQN0385AE01 - HLA-DQ2.5:DQ2.5-glia-γ2) and 8W86 (DQN0385AE02 - HLA-DQ2.5:DQ2.5-hor-3a).

### Generation of mouse major histocompatibility complex (MHC) II knockout and HLA-DQ2.5 transgenic mice (DQ2.5 Tgm)

A *H2-Ab1* gene knockout mouse strain, C57BL/6J-*H2-Ab1*^*em1Csk*^, was established by gene editing using two pairs of Zinc Finger Nucleases (ZFNs) (Sigma-Aldrich) designed to target 5’ upstream and 3’ downstream of *H2-Ab1* gene encoding MHC II in mice. ZFN-target sequence of 5’ upstream and 3’ downstream were CTCTACATCAGGGTGCCAG tgtgg ACCCTGGAATGCTGCAGTT and CCCAACTTAGTCTTC ttttg TTGCTGTTGTAAGAACGCA, respectively (capital letters indicate ZFN binding site and lowercases letters indicate cutting site). HLA-DQ2.5 transgenic mice were kind gifts from Prof. Fugger in University of Oxford, England^28^. HLA-DQ2.5 transgenic mice were crossed with *H2-Ab1* gene knockout mice to establish a *H2-Ab1* KO/*HLA-DQ2.5* transgenic mouse strain. All animal experiments were performed in the specific pathogen-free (SPF) facility in Chugai Pharmaceutical co. ltd.

### Single dose pharmacokinetics study in DQ2.5 Tgm

Male DQ2.5 Tgm (*H2-Ab1* KO/*HLA-DQ2.5* transgenic mouse) were housed (1 mouse/individual cage) in a temperature- and humidity-controlled, and specific pathogen-free room with water, and GFD (AIN-93G, Oriental Yeast) or GCD (CR-LPF, Oriental Yeast) under a 12-h light/12-h dark cycle. The mice were euthanized at the end of the study by CO_2_ inhalation. Eight weeks old animals were randomly categorized into groups based on body weight. In the intravenous study, a single dose of 0.1, 0.5, 2, and 10 mg/kg of DONQ52 or DQN0139 was intravenously administered, and in the subcutaneous study, a single dose of 2 mg/kg of DONQ52 was subcutaneously administered to DQ2.5 Tgm. To determine the plasma antibody concentrations (sparse sampling of *n* = 8 mice/group and *n* = 4 mice/sampling point), blood samples were collected at 5 min, 2 h, 7 h, 24 h, 2 days, 3 days, 7days, and 10 days after antibody dosing. Until 2 days after dosing, approximately 30 μL of blood samples were collected at each sampling points, and from 3days after dosing, approximately 70 μL of blood samples were collected at each sampling points ELISA 96-well plates were precoated with human IgG-heavy and light chain monkey-adsorbed antibody (Bethyl Laboratories, Inc.) and appropriately blocked. Antibody was detected by biotinylated anti-human IgG-Fc monoclonal antibody (clone YG55, generated in house, recognizing delta-GK-amide modified IgG heavy chain constant region that was applied to DONQ52 and DQN0139) and streptavidin-HRP conjugate (Stereospecific Detection Technologies) using TMB substrate. Antibody concentration was measured by Microplate reader: iD3 (Molecular Devices, LLC) and SoftMax Pro 6.4 (Molecular Devices, LLC). Pharmacokinetic parameters were calculated using Phoenix WinNonlin (version 6.4, Certara LP) (Analysis Method: non-compartmental analysis (NCA), model type: plasma, dose options: extravascular or intravenous bolus, calculation method: linear log trapezoidal). Time-points for T_1/2_ calculation were automatically set by WinNonlin based on the time profile in plasma concentrations. For calculation of the PK parameters, BLQ values were treated as 0 μg/mL. The PK parameters were calculated using the theoretical dose level. Bioavailability (F) was calculated in Microsoft Excel 2016 using the following formula: F (%) = (AUCinf/dose) at subcutaneous dosing/(AUCinf/dose) at intravenous dosing × 100. All pharmacokinetic parameters of DONQ52 were evaluated using the concentrations of DONQ52 in plasma excluding anti-drug antibody-positive samples. Calculated numerical pharmacokinetic parameters are described in Supplementary Table 10.

### In vivo antigen immunization study in DQ2.5 Tgm

Male DQ2.5 Tgm (*H2-Ab1* KO/*HLA-DQ2.5* transgenic mouse) were co-housed (3 or 4 mice/individual cage) in a temperature- and humidity-controlled, and specific pathogen-free room fed with a GFD (AIN-93G, Oriental Yeast) under a 12-h light/12-h dark cycle. Six weeks old animals were randomly categorized into 4 groups for uniform body weight by JMP (version 11.2.1, JMP Statistical Discovery LLC.; Block Allocation, Levene’s test) just before experiment. For the 33mer gliadin peptide immunization, except for non-treated group (*n* = 10), DQ2.5 Tgm were administered Control Ab (anti-KLH antibody) (*n* = 10), DQN0139 (*n* = 10) or DONQ52 (*n* = 10) intraperitoneally three times a week from day 0. For the KLH immunization, except for non-treated group (*n* = 10), DQ2.5 Tgm were administered vehicle (PBS) (*n* = 10), DQN0139 (*n* = 10) or DONQ52 (*n* = 10) intraperitoneally three times a week from day 0. PBS was used as a solvent for antibody dilution. These mice, except for the non-treated group, were subcutaneously immunized with antigen (100 μg of 33mer gliadin peptide per head, or 10 μg of KLH) using Complete Freund’s adjuvant (CFA) as an adjuvant on day 2. The details of each group are described in Supplementary Table 11. Seven days after immunization, mice were euthanized under isoflurane-induced anesthesia for all blood draws and spleen sampling. Isolated splenocytes were distributed individually on mouse IL-2 ELISPOT plates (Mabtech, 4 × 10^6^ cells/well for the 33mer gliadin peptide immunization, 2.5 × 10^6^ cells/well for KLH immunization), stimulated with antigen used for immunization (final concentration, 33mer gliadin peptide: 50 μM, KLH: 20 μg/mL) or culture medium alone, and cultured overnight in incubators (set at 37 °C and 5% CO_2_). Each condition had 3 wells (triplicate). Spot detection was performed according to the manufacturer’s protocol. After spots detection, ELISPOT plates were dried and spot counts were evaluated as SFC using ImmunoSpot S6 ENTRY (Software version: ImmunoSpot 5.1.36). Blood was collected on day 2, 4, 7, and 9. Blood samples were centrifuged, and plasma was collected to measure antibody concentration. Method of antibody concentration measurement is as described in above section (Single dose pharmacokinetics study in DQ2.5 Tgm). Statistical analysis was performed using JMP (version 15.0.0). The significance level was set to 5% by two-side test. First, student’s *t*-test was performed to compare SFC between Non-treated group and Control Ab treatment group or Vehicle group. Next, two-way ANOVA, Dunnett’s test as multiple comparison test was performed to compare SFC of Control Ab treatment group or Vehicle group to DQN0139 treatment group, DONQ52 treatment group.

### In vivo DQ2.5-glia-γ2 peptide immunization study in DQ2.5 Tgm

Male DQ2.5 Tgm (*H2-Ab1* KO/*HLA-DQ2.5* transgenic mouse) were co-housed (3 or 4 mice/individual cage) in a temperature- and humidity-controlled, and specific pathogen-free room fed with a GFD (AIN-93G, Oriental Yeast). Six weeks old animals were randomly categorized into 3 groups based on uniform body weight by JMP just before experiment. For the DQ2.5-glia-γ2 peptide immunization group, DQ2.5 Tgm were implanted with pumps which were filled with Control Ab (13.23 mg/mL) (*n* = 10) or DONQ52 (4.50 mg/mL) (*n* = 10). These mice, except for the non-treated group (*n* = 10), were subcutaneously immunized with antigen (10.0 μg of DQ2.5-glia-γ2 peptide per head) using Complete Freund’s adjuvant (CFA) as an adjuvant on day 7. The details of each group are described in Supplementary Table 11. Seven days after immunization, were euthanized under isoflurane-induced anesthesia for all blood draws and spleen sampling. Isolated splenocytes were distributed individually on mouse IL-2 ELISPOT plates (Mabtech, 4 × 10^6^ cells/well), stimulated with DQ2.5-glia-γ2 peptide (final concentration, 50 μM) or culture medium alone, and cultured overnight in incubators (set at 37 °C and 5% CO_2_). Each condition had 3 wells (triplicate). Spot detection was performed according to the manufacturer’s protocol. After spot detection, ELISPOT plates were dried, and spot counts were evaluated as SFC using ImmunoSpot S6 ENTRY (Software version: ImmunoSpot 5.1.36). Blood was collected on day 7 and 9. Blood samples were centrifuged, and plasma was collected to measure antibody concentration. The method of antibody concentration measurement is as described in above section (Single dose pharmacokinetics study in DQ2.5 Tgm). Statistical analysis was performed using JMP (version 15.0.0). The significance level was set to 5% by two-side test. Two-tailed unpaired student’s t-test was performed to compare SFC of Control Ab treatment group and that of DONQ52 treatment group.

### Statistical analysis

JMP version 11.2.1 or 15.0.0 (JMP Statistical Discovery LLC.) and Microsoft Office Excel 2016 and Prism 7.0 software, Prism 7.0 software (GraphPad Software, Inc.) were used for statistical analysis. The details of the statistical tests carried out are indicated in the respective figure legends and method. The error bars in the figures represent the 95% CI or SD. In in vivo study, Mice were randomized into different groups before experiments.

### Reporting summary

Further information on research design is available in the Nature Portfolio Reporting Summary linked to this article.

### Supplementary information

Supplementary Information

Peer Review File

Reporting Summary

### Source data

Source Data

## Data Availability

The structure data generated in this study have been deposited in the Protein Data Bank (PDB) under the accession code 8W83, 8W84, 8W85, and 8W86. The structural data used in this study are available in the Protein Data Bank (PDB) under accession code 5KSA, 1S9V, 6XP6, 4OZH, 4OZF, 4OZG. The flowcytometry data generated in this study have been deposited in the FlowRepository database under accession code FR-FCM-Z7YG corresponding to Fig. 2c. The other source data generated in this study are provided in the Supplementary Information and the Source Data file. Other materials, including mAb sequence and protein information, can be provided to academic researchers for free via a material transfer agreement (MTA) that includes a research plan. Source data are provided with this paper.
