# Supplementary material for: Characterizations of a neutralizing antibody broadly reactive to multiple gluten peptide:HLA-DQ2.5 complexes in the context of celiac disease
Source: Nat Commun. 2023 Dec 22;14:8502. doi: 10.1038/s41467-023-44083-4 (PMC10746718; doi:10.1038/s41467-023-44083-4)
Supplement: Supplementary file 1 — Supplementary Information [file 41467_2023_44083_MOESM1_ESM.pdf]

Supplemental information for

Characterizations of a neutralizing antibody broadly reactive to multiple gluten peptide:HLA-DQ2.5 complexes in the context of celiac disease

Yuu Okura[1], Yuri Ikawa-Teranishi[2], Akihiko Mizoroki[2], Noriyuki Takahashi[3], Takashi Tsushima[3], Machiko Irie[2], Zulkarnain Harfuddin[3], Momoko Miura-Okuda[3], Shunsuke Ito[4], Genki Nakamura[2], Hiroaki Takesue[2], Yui Ozono[2], Masamichi Nishihara[2], Kenta Yamada[2], Gan Siok Wan[3], Akira Hayasaka[2], Shinya Ishii[2], Tetsuya Wakabayashi[2], Masaru Muraoka[2], Nishiki Nagaya[2], Hiroshi Hino[2], Takayuki Nemoto[4], Taichi Kuramochi[2], Takuya Torizawa[2], Hideaki Shimada[3], Takehisa Kitazawa[2], Makoto Okazaki[3], Junichi Nezu[5], Ludvig M. Sollid[6,7] & Tomoyuki Igawa[1]*

1 Translational Research Division, Chugai Pharmaceutical Co., Ltd., Tokyo, Japan.
2 Research Division, Chugai Pharmaceutical Co., Ltd., Kanagawa, Japan.
3 Chugai Pharmabody Research Pte. Ltd., 3 Biopolis Drive, Singapore.
4 Translational Research Division, Chugai Pharmaceutical Co., Ltd., Kanagawa, Japan.
5 R&D Portfolio Management Department, Chugai Pharmaceutical Co., Ltd., Tokyo, Japan.
6 Department of Immunology, Oslo University Hospital, Oslo, Norway.
7 Institute of Clinical Medicine, University of Oslo, Oslo, Norway.

*Corresponding Author; igawatmy@chugai-pharm.co.jp

Supplementary Fig. 1 to Fig. 10
Supplementary Table 1 to Table 11
Supplementary References

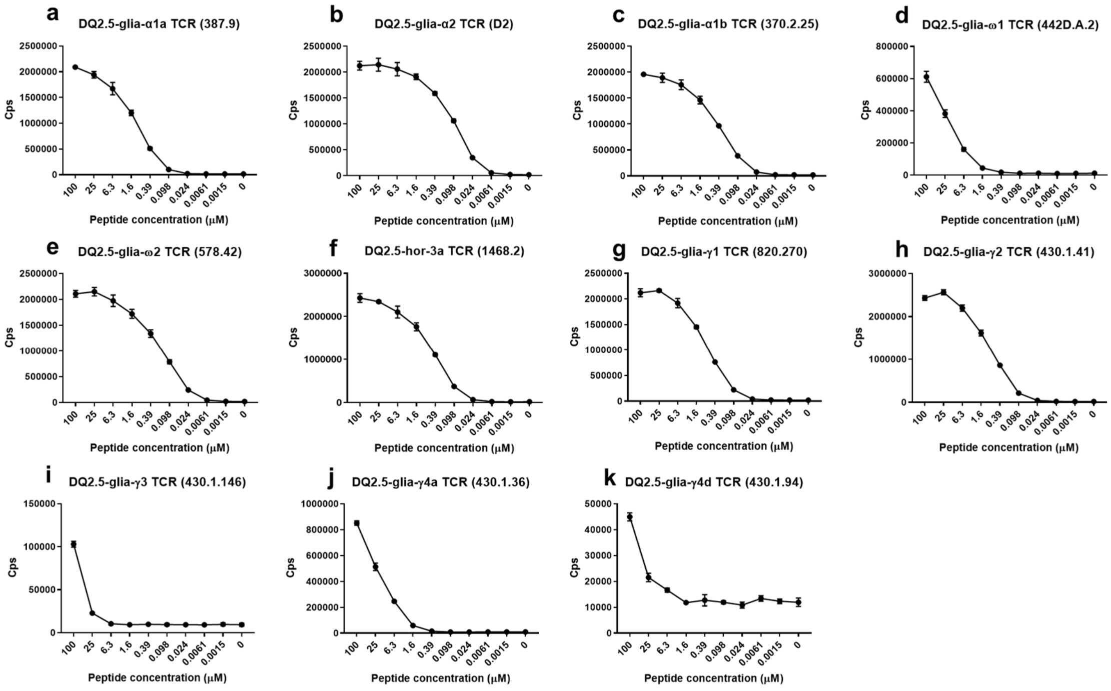

Supplementary Fig. 1: Schematics of insert sequence strategies of HLA-DR, HLA-DP and HLA-DQ molecules

Activation of (**a**) DQ2.5-glia-α1a epitope, (**b**) DQ2.5-glia-α2 epitope, (**c**) DQ2.5-glia-α1b epitope, (**d**) DQ2.5-glia-ω1 epitope, (**e**) DQ2.5-glia-ω2 epitope, (**f**) DQ2.5-hor-3a epitope, (**g**) DQ2.5-glia-γ1 epitope, (**h**) DQ2.5-glia-γ2 epitope, (**i**) DQ2.5-glia-γ3 epitope, (**j**) DQ2.5-glia-γ4a epitope, (**k**) DQ2.5-glia-γ4d epitope specific TCR expressed αβTCR-knockout Jurkat-NFAT-Luc2 (2.0×10^4 cells/well) by co-culturing with IHW09023 (8.0×10^4 cells/well) in the presence of serially diluted corresponding peptide (Supplementary Table 6). After overnight culture, 50 µL of cultured cells were harvested and mixed with 50 uL of Bio-Glo, and then incubated at room temperature for 10 minutes. Luminescence count per second (cps) was measured with EnVision, followed by analysis using Outlook Excel 2013 (Microsoft Corporation) and GraphPad Prism software (GraphPad Software, Inc.). Data are from an assay performed in triplicate (n=3) and are plotted as mean ± SD.

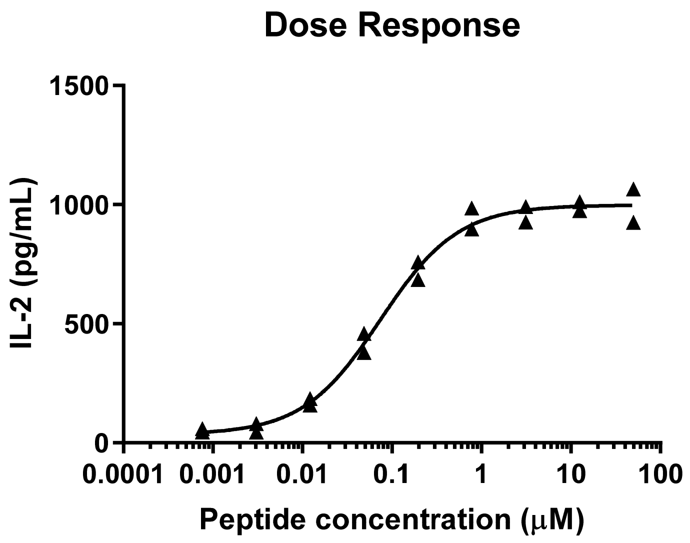

Supplementary Fig. 2: 33mer gliadin peptides mediated IL-2 production by using human $CD4^+$ T cells expressing TCRs specific for DQ2.5-glia-α2

IL-2 production of DQ2.5-glia-α2 epitope specific TCR expressed human $CD4^+$ T cells (1.0×10^5 cells/well) by co-culturing with IHW09023 (4.0×10^5 cells/well) with anti-human MHC Class I antibody (Clone: W6/32, Bio X Cell, Inc), anti-HLA-DR antibody (Clone: L243, Bio X Cell, Inc) in the presence of serially diluted 33mer gliadin peptide. After overnight culture, supernatants were collected to measure IL-2 concentration, followed by analysis using Outlook Excel 2013 (Microsoft Corporation) and GraphPad Prism software (GraphPad Software, Inc.). Data are from an assay performed in duplicate (n=2) and are plotted as an individual, and nonlinear fitting.

a b c d
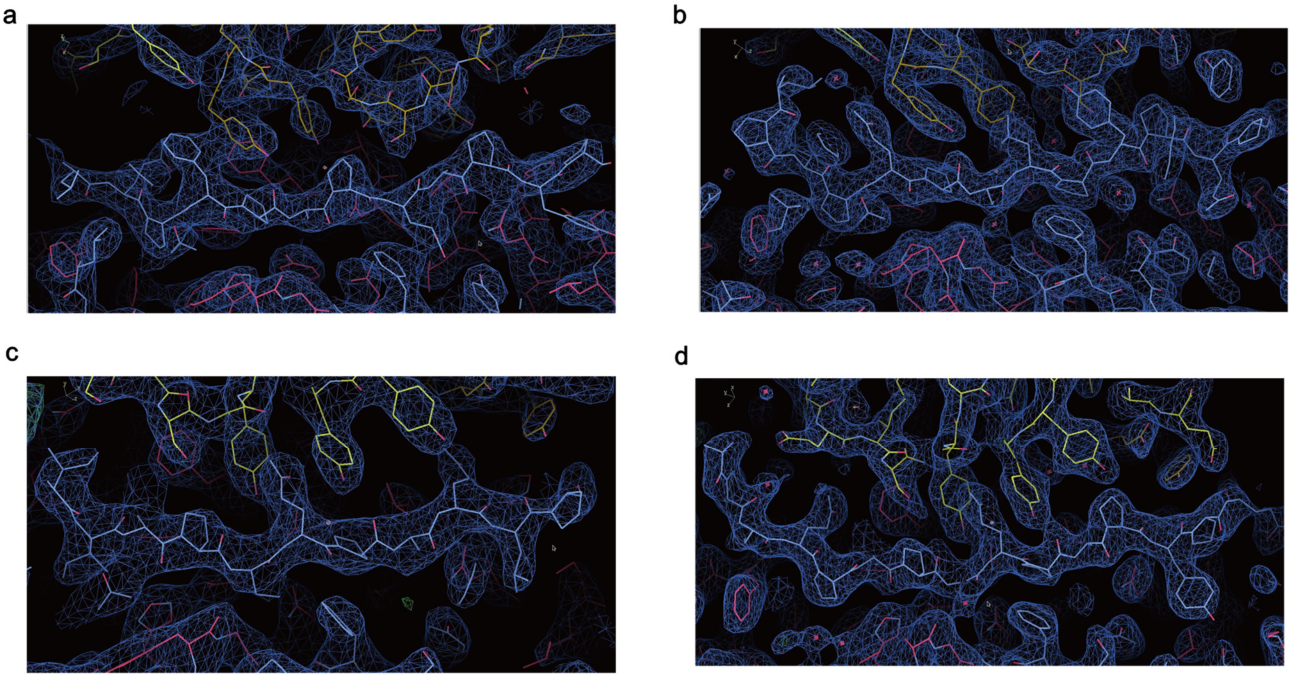

Supplementary Fig. 3: Electron density maps of the regions of epitope/paratope interactions

Electron density maps of the regions of (a) the DQN0344AE02 with HLA-DQ2.5:DQ2.5-glia-α1a interaction, (b) the DQN0344AE02 with HLA-DQ2.5:DQ2.5-glia-α2 interaction,. (c) the DQN0385AE01 with HLA-DQ2.5:DQ2.5-glia-γ2 interaction, and (d) the DQN0385AE02 with HLA-DQ2.5: DQ2.5-hor-3a interaction. Light yellow-, dark yellow-, red- and blue-colored residues fitted on the electron density maps, respectively, represent L-chain of Fabs, H-chain of Fabs, HLA-DQA1*05:01, and HLA-DQB1*02:01 with the loaded peptides.

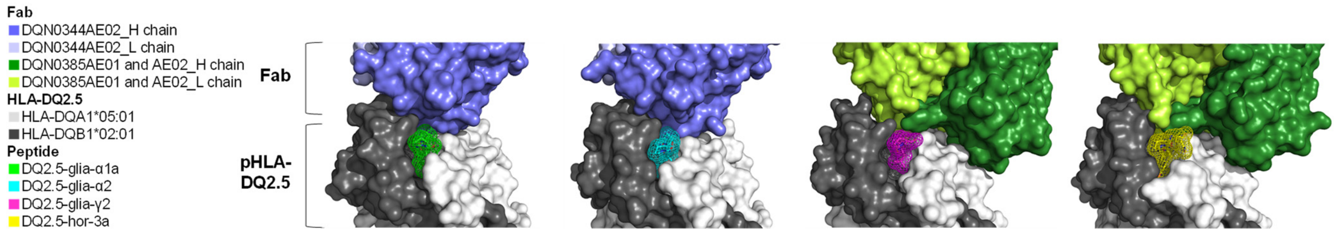

	DQN0344AE02 - HLA-DQ2.5:DQ2.5-glia-α1a	DQN0344AE02 - HLA-DQ2.5:DQ2.5-glia-α2	DQN0385AE01 - HLA-DQ2.5:DQ2.5-glia-γ2	DQN0385AE02 - HLA-DQ2.5:DQ2.5-hor-3a
Total BSA ($Å^2$)	1,875	2,196	2,479	2,661
Contribution of VH (%)	89.0	77.1	39.7	39.8
Contribution of VL (%)	11.0	22.9	60.3	60.2
Interface with HLA-DQA (%)	35.0	37.7	57.3	54.7
Interface with HLA-DQB (%)	37.9	28.1	16.1	16.5
Interface with peptide (%)	27.1	34.2	26.6	28.7

	3.C11 - HLA-DQ2.5:DQ2.5-glia-α2	S16 TCR - HLA-DQ2.5:DQ2.5-glia-α2	JR5.1 TCR - HLA-DQ2.5:DQ2.5-glia-α2	D2 TCR - HLA-DQ2.5:DQ2.5-glia-α2
Total BSA ($Å^2$)	2,384	2,064	1,716	2,090
Contribution of VH (%)	72.0	60.6	72.0	63.0
Contribution of VL (%)	28.0	39.4	28.0	37.0
Interface with HLA-DQA (%)	50.1	33.1	37.6	33.4
Interface with HLA-DQB (%)	25.8	44.8	38.9	44.0
Interface with peptide (%)	24.1	22.1	23.5	22.6

Supplementary Fig. 4: Buried Surface Area (BSA) of Fabs and TCRs for their interaction with various gluten pHLA-DQ2.5

3.C11 - HLA-DQ2.5:DQ2.5-glia-α2 complex (PDB ID_6XP6), S16 TCR - HLA-DQ2.5:DQ2.5-glia-α2 complex (PDB ID_4OZH), JR5.1 TCR - HLA-DQ2.5:DQ2.5-glia-α2 complex (PDB ID_4OZF), D2 TCR - HLA-DQ2.5:DQ2.5-glia-α2 complex (PDB ID_4OZG).

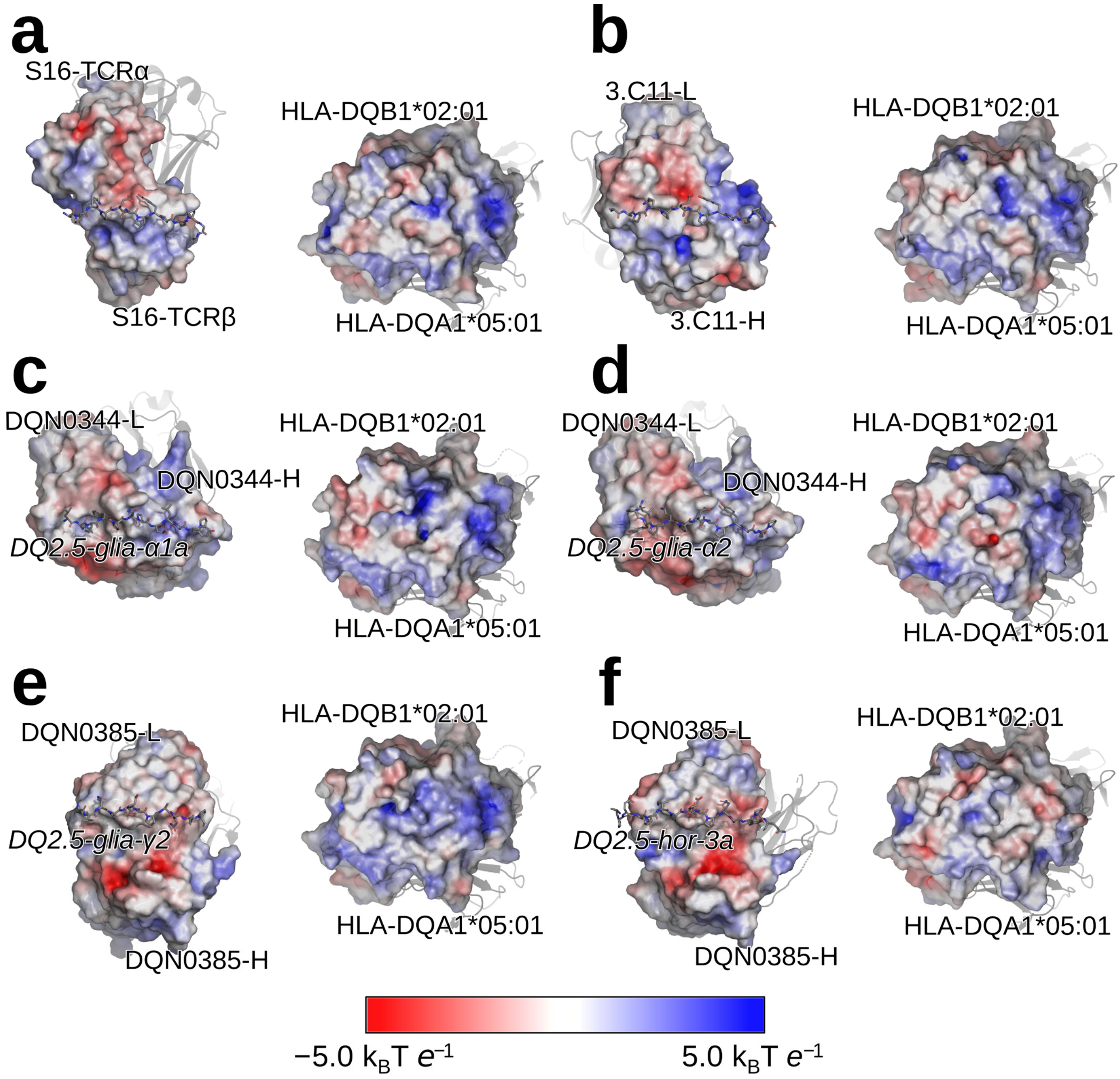

Supplementary Fig. 5: Electrostatic surface potential (ESP) of Fabs, TCRs, and various gluten p-HLA-DQ2.5

Each electrostatic surface potential (colored as indicated in the figure) in antibodies and pHLA-DQ2.5 of (a) the S16 - HLA-DQ2.5:DQ2.5-glia-α2 complex (PDB ID_4OZH), (b) the 3.C11 - HLA-DQ2.5:DQ2.5-glia-α2 complex (PDB ID_6XP6), (c) the DQN0344AE02 - HLA-DQ2.5:DQ2.5-glia-α1a complex, (d) the DQN0344AE02 - HLA-DQ2.5:DQ2.5-glia-α2 complex, (e) the DQN0385AE01 - HLA-DQ2.5:DQ2.5-glia-γ2 complex and (f) the DQN0385AE02 - HLA-DQ2.5:DQ2.5-hor-3a complex. Surface potentials were generated with PyMOL version 2.3.

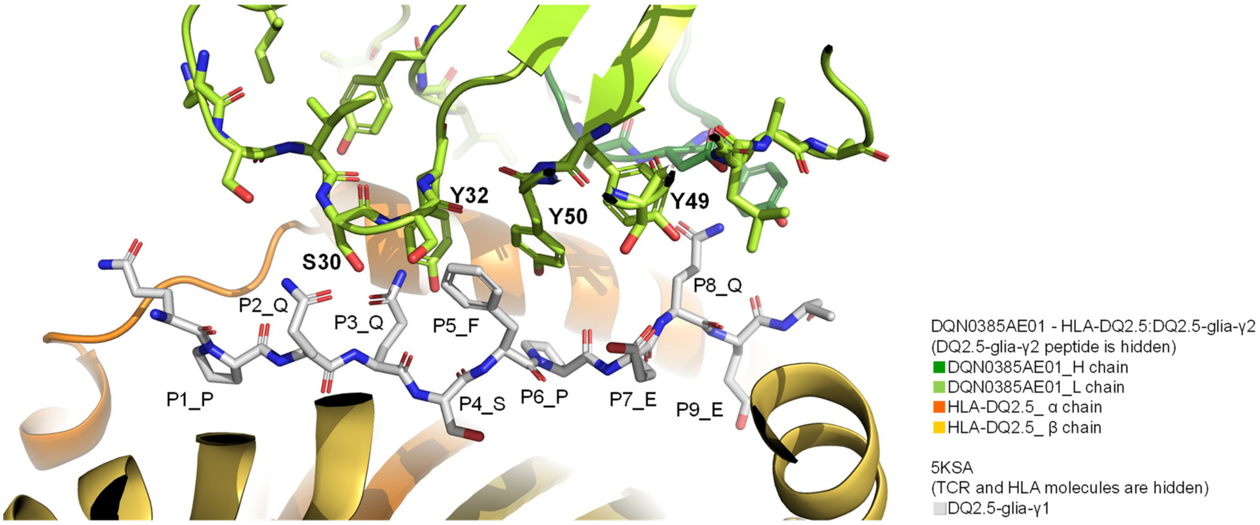

Supplementary Fig. 6: Superimposing of DQ2.5-glia-γ1 peptide structure from complex with TCR and HLA (PDB ID:5KSA) on DQN0385AE01 - HLA-DQ2.5:DQ2.5-glia-γ2 complex structure

Superimposed structures DQN0385AE01 - HLA-DQ2.5:DQ2.5-glia-γ2 and PDB_5KSA (Bel602-DQ8.5-glia-γ1 complex). DQN0385AE01 and HLA-DQ2.5 of the former structure, and DQ2.5-glia-γ1 peptide of the latter structure are displayed and colored as described in the figure. DQ2.5-glia-γ2 peptide of the former structure, and Bel602 TCR and HLA-DQ8.5 proteins of the latter structure are hidden from these superimposed structures.

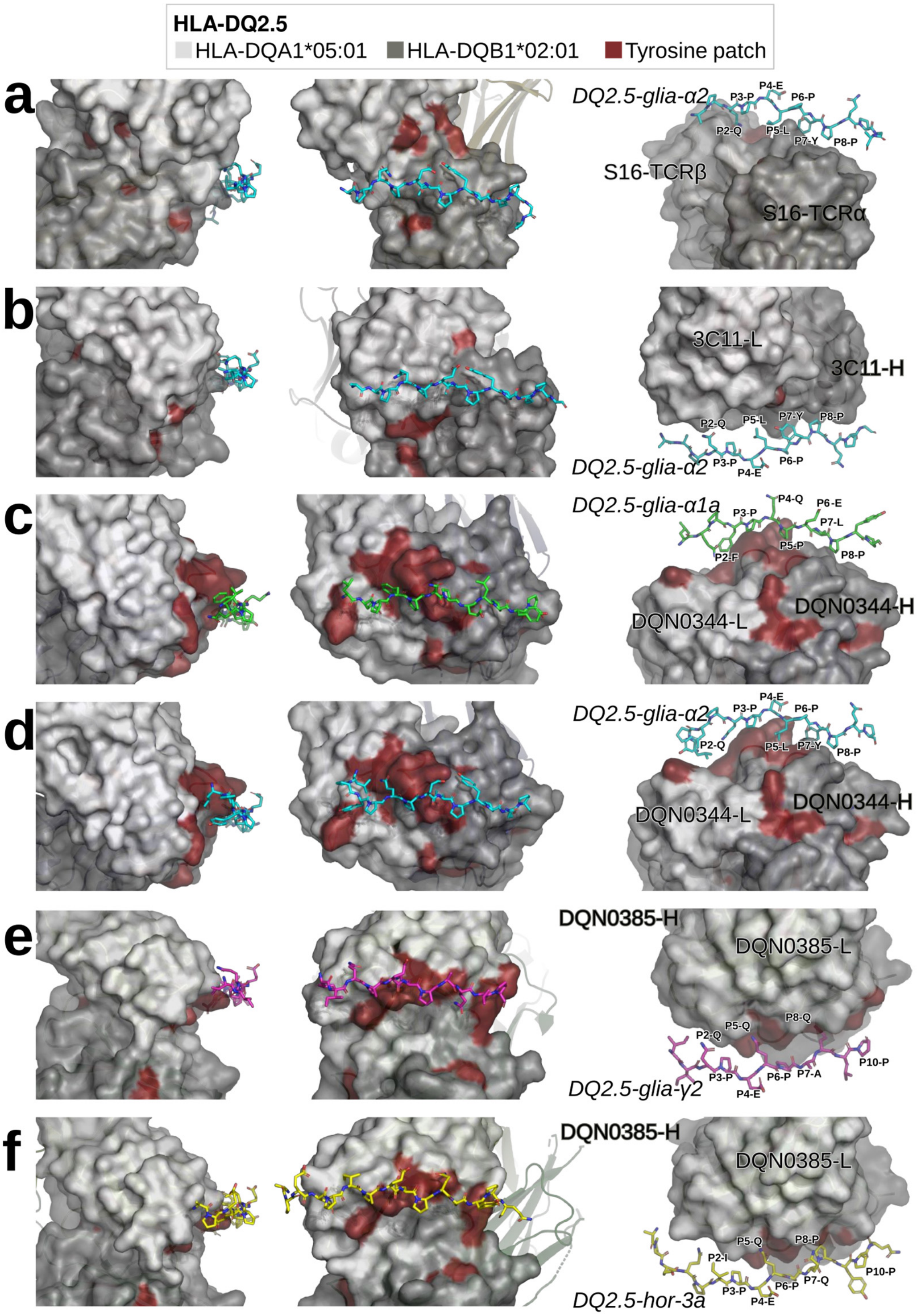

Supplementary Fig. 7: Surface representation illustrating interactions of TCRs or Fabs with various gluten pHLA-DQ2.5

A series of protuberance structures formed by Tyr residues are present in Fab DQN0344 and Fab DQN0385 while they are not observed in the S16-TCR and 3.C11 antibody structures. Surface representation illustrating (a) the S16-TCR with DQ2.5-glia-α2 (PDB ID_4OZH), (b) the 3.C11 Fab with DQ2.5-glia-α2 (PDB ID_6XP6), (c) DQN0344AE02 Fab with DQ2.5-glia-α1a, (d) DQN0344AE02 Fab with DQ2.5-glia-α2, (e) DQN0385AE01 Fab with DQ2.5-glia-γ2 and (f) DQN0385AE02 Fab with DQ2.5-hor-3a. Tyr residues are represented by red color patches, and peptide are colored as indicated. Surface representations were generated with PyMOL version 2.3.

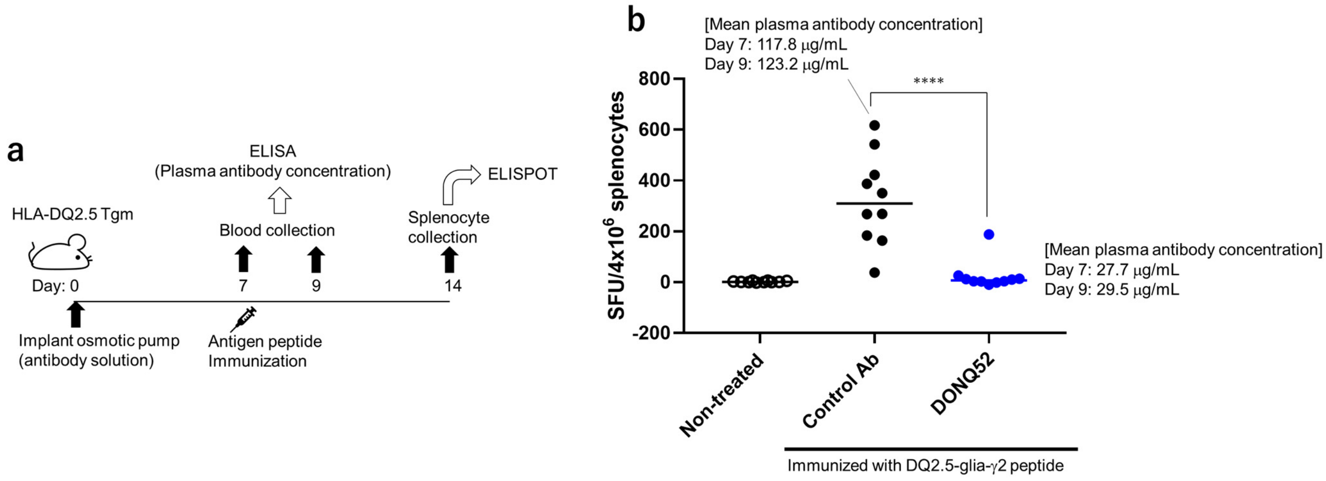

Supplementary Fig. 8: In vivo neutralizing activity of DONQ52 against DQ2.5-glia-γ2 in DQ2.5 Tgm

Assessment of ability of DONQ52 to block HLA-DQ2.5 restricted T cell response to DQ2.5-glia-γ2 in vivo using an IL-2 ELISPOT assay on splenocytes of DQ2.5 Tgm immunized with the DQ2.5-glia-γ2 peptide. (a) Outline of experiment. On day 0, DQ2.5 Tgm were implanted with osmotic pumps that delivered DONQ52 (n=10) or control Ab (anti-KLH) (n=10). Mice in a non-treated group (n=10) received no antibody. Antibody plasma concentration of DONQ52 was 27.7μg/mL on day 7 and 29.5μg/mL on day 9. The mice, except those in the non-treated group, were subcutaneously immunized on day 7 with DQ2.5-glia-γ2 (10 μg per mouse) using CFA as adjuvant. Splenocytes isolated on 7 days after immunization were subjected to IL-2 ELISPOT assay using 50 μM of DQ2.5-glia-γ2 peptide or sham as antigen. b) IL2 ELISPOT assay. Results are presented as spot forming cells (SFC) / 4 × 10^6 splenocytes. SFC with sham stimulation are subtracted as background from SFC of DQ2.5-glia-γ2 peptide stimulation. Mean spot counts of each animal (triplicate wells) with horizontal bars representing median spot count of each group are shown. Points: mean spot count of each animal (triplicate well), bars: median spot count of each group. ****P < 0.0001 statistical significance for the differences was determined by two-tailed unpaired student's t test.

	Efficacy	Pharmacokinetics	Safety
**Anti-HLA-DQ mAbs**	Efficacy is expected, since mAb blocks all gluten epitopes	Poor PK due to abundant expression of HLA II, leading insufficient mAb exposure with realistic administration dose	・Risk of immunogenicity<br>・Risk of infection<br>・Negative effect on vaccine<br>・Risk of disrupting thymic T cell education
**Anti-gluten pHLA-DQ2.5 specific mAbs**	Efficacy would be limited, since mAb only blocks certain pathogenic gluten epitopes	Favorable PK due to limited target molecular amount, leading enough mAb exposure with realistic administration dose	Less concern on immunogenicity, infection, vaccination, and thymic T cell education
**DONQ52**	Efficacy is expected, since mAb blocks all pathogenic gluten epitopes, as far as Pro/Gln motif is relevant to pathogenic gluten epitopes	Favorable PK due to limited target molecular amount, leading enough mAb exposure with realistic administration dose	Less concern on immunogenicity, infection, vaccination, and thymic T cell education

Supplementary Fig. 9: Distinct advantage of DONQ52 to anti-HLA-DQ antibodies and anti-gluten pHLA-DQ2.5 specific antibodies

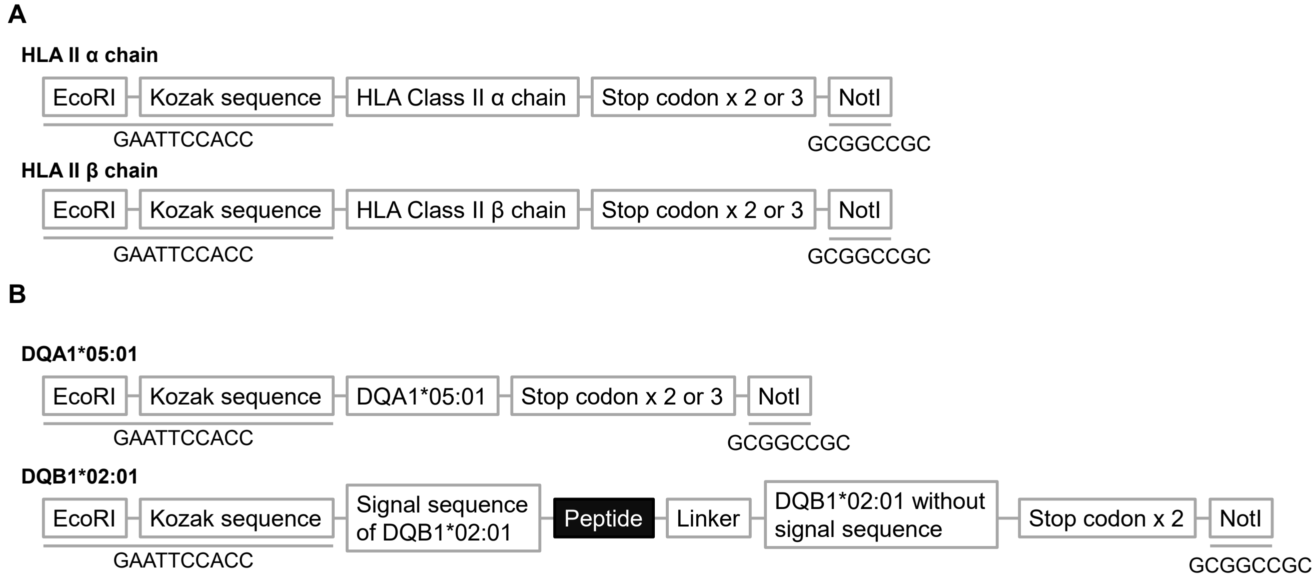

Supplementary Fig. 10: Schematics of insert sequence strategies of HLA-DR, HLA-DP and HLA-DQ molecules

(**A**) Schematic drawing of pHLA-DQ2.5 expressed by Ba/F3 cells. (**B**) Schematic drawing of HLA-DR, HLA-DP and HLA-DQ molecules expressed by Ba/F3 cells. A factor X cleavage site is used as a linker between peptide and DQB1*02:01.

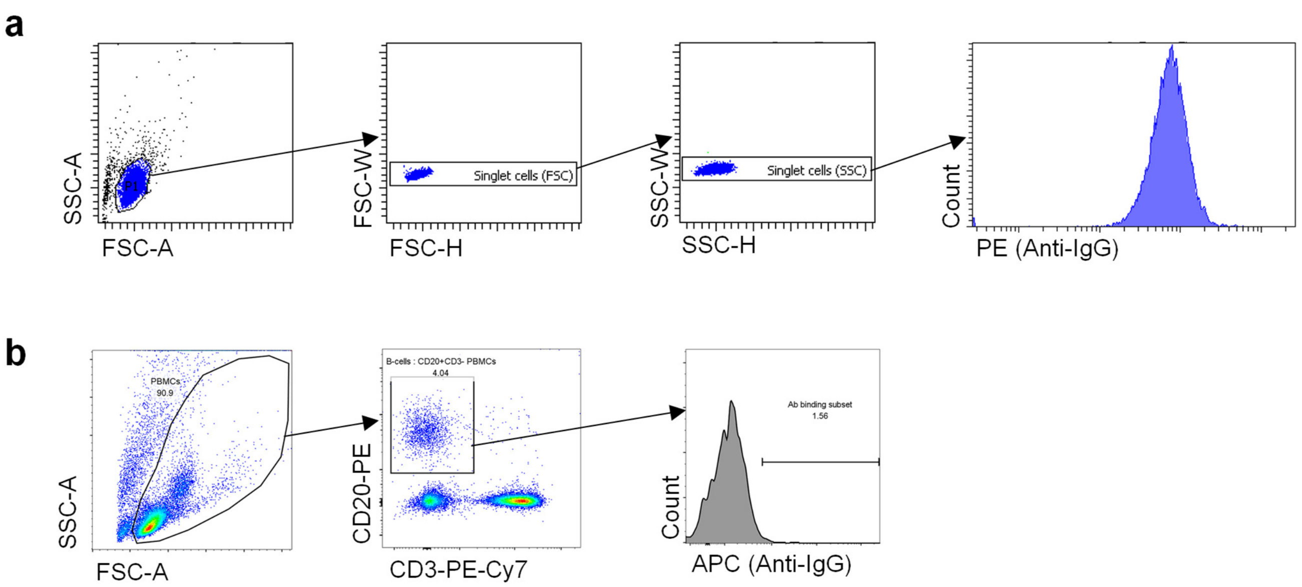

Supplementary Fig. 11: Example of gating strategies used for FACS experiments

(**a**) Example of gating strategy to determine the antibody binding MFI to Ba/F3 cells expressing various pHLA-DQ2.5 or non-HLA-DQ2.5 (Fig.1a, Fig.2a) (**b**) Example of gating strategy to determine the antibody binding MFI to Ba/F3 cells expressing various pHLA-DQ2.5 or non-HLA-DQ2.5 (Fig.2c)

Supplementary Table 1: Amino acid sequence of gluten peptides used as immunogen, or in vitro, in vivo studies

Peptide name	Epitope	Peptide sequence
**33mer gliadin**	DQ2.5-glia-α1a, DQ2.5-glia-α2, DQ2.5-glia-α1b	LQLQPFPQPELPYPQPELPYPQPELPYPQPQPF
**ω1/2 gliadin**	DQ2.5-glia- ω1, DQ2.5-glia-ω2	EQPFPQPEQPFPWQP
**B/C hordein**	DQ2.5-hor-3a	EPEQPIPEQPQPYPQQ
**γ1 gliadin**	DQ2.5-glia-γ1	PQQPQQSFPEQEQPA
**γ2 gliadin**	DQ2.5-glia-γ2	GQGIIQPEQPAQLIR
**γ3 gliadin**	DQ2.5-glia-γ3	EQPFPEQPEQPYPEQPEQPFPQP
**γ4a gliadin**	DQ2.5-glia-γ4a	FSQPEQEFPQPQ
**γ4d gliadin**	DQ2.5-glia-γ4d	WPQQQPFPQPEQPFCEQPQR

List of pathogenic gluten peptides with amino acid sequence and contained epitopes, used as an immunogen, or in vitro, in vivo studies.

Supplementary Table 2: Amino acid sequence of the peptides linked with DQB1*02:01 and expressed by Ba/F3 cells

Peptide name	Epitope in peptide	Peptide sequence
**33mer gliadin**	**DQ2.5-glia-α1a, DQ2.5-glia-α2, DQ2.5-glia-α1b**	**LQLQPFPQPELPYPQPELPYPQPELPYPQPQPF**
29mer hCLIP	-	KLPKPPKPVSKMRMATPLLMQALPMGALP
HBV1	-	PDRVHFASPLHVAWR
Salmonella	-	MMAWRMMRY
M.bovis	-	KPLLIIAEDVEGEY
Thyroid peroxidase	-	YIDVWLGGLAENFLPY
**α1 gliadin**	**DQ2.5-glia-α1a**	**QPFPQPELPYP**
**α2 gliadin**	**DQ2.5-glia-α2**	**FPQPELPYPQP**
γ1 gliadin	DQ2.5-glia-γ1	QPQQSFPEQQP
γ2 gliadin	DQ2.5-glia-γ2	GIIQPEQPAQLP
**ω1 gliadin**	**DQ2.5-glia-ω1**	**QPFPQPEQPFP**
**ω2 gliadin**	**DQ2.5-glia-ω2**	**FPQPEQPFPWQ**
**B/C hordein**	**DQ2.5-hor-3a**	**PQQPIPEQPQPYPQQP**
α3 gliadin	DQ2.5-glia-α3	PFRPEQPYPQP
α1b gliadin	DQ2.5-glia-α1b	LPYPQPELPYP
γ4a gliadin	DQ2.5-glia-γ4a	FSQPEQEFPQP
γ4b gliadin	DQ2.5-glia-γ4b	FPQPEQEFPQP
avenin 1	DQ2.5-ave-1a	QPYPEQEEPFV
avenin 2	DQ2.5-ave-1b	QPYPEQEQPFV
hordein 1	DQ2.5-hor-1	PQQPFPQPEQPFRQ
hordein 2	DQ2.5-hor-2	QEFPQPEQPFPQQP
secalin 1	DQ2.5-sec-1	PEQPFPQPEQPFPQ
secalin 2	DQ2.5-sec-2	QPFPQPEQPFPQSQ
glutenin 1	DQ2.5-glut-L1	QPPFSEQEQPVLP
14mer 1	-	PQQQTLQPEQPAQLP
α1a homologue	-	QPFPQPELPFP
α2 homologue	-	FPQPELPFPQP
ω1 homologue	-	QPFPQPEQPIP
ω2 homologue	-	FPQPEQPIPVQ
W04	-	QPFPQPEQPIPVQ
W06	-	LQPFPQPELPFPQP
W26	-	PFPLQPEQPFPQP
avenin3	DQ2.5-ave-1c	QPYPEQEQPIL
26mer gliadin	DQ2.5-glia-γ3, DQ2.5-glia-γ4c, DQ2.5-glia-γ5	FLQPEQPFPEQPEQPYPEQPEQPFPQ

List of peptides linked with DQB1*02:01 expressed by Ba/F3 cells with amino acid sequence. Epitope name was followed nomenclature of CeD–relevant gluten epitopes recognized by $CD4^{+}$ T cells, bold indicates peptides with immune dominant epitopes (1), underline indicates identified peptide-binding registers (2).

Supplementary Table 3: Binding profile of antibodies against pHLA-DQ2.5 expressing Ba/F3 cell lines

Cell lines (abbreviation)	Relative MFI† (%)		MFI							
			DQN0139		Control Ab		DONQ52		FACS Buffer	
	Mean	SD	Mean	SD	Mean	SD	Mean	SD	Mean	SD
- *	-0.5	1.9	4177	11	192	35	171	74	6	1
hCLIP	2.4	0.8	20654	263	23	13	517	175	4	1
HBV1	-0.2	0.5	863	4	12	8	10	4	4	1
Salmonella	-1.3	0.4	761	45	20	9	10	3	4	1
M.bovis	-0.1	0.1	7357	289	22	15	14	7	4	0
TPO	-0.2	0.1	14646	247	42	25	11	8	4	0
33mer gliadin	86.2	2.8	33972	126	53	32	29297	953	8	4
α1 gliadin	122.4	2.3	6790	25	19	17	8308	153	5	0
α2 gliadin	74.7	1.7	4250	60	13	3	3178	73	4	0
γ1 gliadin	85.0	5.5	2660	49	26	12	2265	145	4	0
γ2 gliadin	7.1	0.6	16231	125	11	10	1160	103	3	1
ω1 gliadin	111.4	3.0	989	18	13	5	1100	29	5	0
ω2 gliadin	54.3	1.3	1235	12	10	3	675	16	5	1
BC hordein	46.2	0.2	5690	147	26	15	2645	14	5	0
α3 gliadin	74.3	2.9	2349	20	14	7	1748	68	5	0
α1b gliadin	99.4	1.9	2368	18	10	3	2354	44	5	1
γ4a gliadin	25.7	0.8	1935	26	10	3	506	15	5	1
γ4b gliadin	67.4	0.7	2966	63	17	12	2003	22	6	1
avenin1	54.2	1.4	5429	65	7	1	2947	75	5	1
avenin2	83.7	2.0	3788	63	13	3	3173	75	5	0
avenin3	78.9	1.7	3545	22	16	7	2801	60	6	1
hordein1	47.2	1.1	1108	26	8	1	528	12	6	1
hordein2	74.9	3.2	1858	67	12	6	1396	60	6	1
secalin1	93.6	3.4	1444	18	11	5	1352	49	6	0
secalin2	84.8	1.8	2757	19	11	1	2340	49	6	1
glutenin1	10.3	0.5	5336	187	17	10	563	29	6	1
14mer1	50.3	5.4	22818	279	15	6	11484	1231	5	0
α1a homologue	111.0	1.8	18609	139	13	2	20661	342	14	16
α2 homologue	84.2	2.4	4271	48	9	6	3597	101	4	0
ω1 homologue	63.0	1.0	2341	10	10	3	1479	23	5	1
ω2 homologue	17.7	0.7	1545	15	14	2	285	11	4	0
W04	65.3	0.7	6362	36	18	5	4160	44	6	1
W06	89.4	2.0	22464	132	17	6	20077	443	4	0
W26	81.7	1.3	5981	99	25	13	4889	80	6	3
26mer gliadin	73.4	0.6	13764	74	15	3	10110	88	6	2

Data are from an assay performed in triplicates (n=3). * Ba/F3 cell line expressing HLA-DQ2.5 with no peptide ligand as part of the construct. † MFI: Mean Fluorescence Intensity. Relative MFI: Relative value of MFI to DQN0139 (anti-HLA-DQ neutralizing antibody).

Supplementary Table 4: Binding profile of DONQ52 against HLA II expressing Ba/F3 cell lines

Cell lines	MFI													
	FACS Buffer		MOPC-173		Control Ab		DONQ52		DQN0139		Tu39		SPV-L3	
	Mean	SD	Mean	SD	Mean	SD	Mean	SD	Mean	SD	Mean	SD	Mean	SD
Ba/F3-HLA-DP	8	0	152	40	11	2	17	7	15	5	22254	818	229	11
Ba/F3-HLA-DR	10	1	227	46	11	2	11	2	19	9	15863	498	214	15
Ba/F3-HLA-DQ8	14	2	811	129	70	8	64	15	28636	181	1628	21	18363	59
Ba/F3-HLA-DQ5.1	12	1	248	31	20	8	19	6	21	5	782	56	1850	116
Ba/F3-HLA-DQ6.3	9	0	164	46	259	53	222	14	312	73	1408	187	17129	336
Ba/F3-HLA-DQ7.5	10	0	156	64	222	53	268	31	5468	144	860	22	3591	352
Ba/F3-HLA-DQ7.3	11	0	326	69	575	233	467	66	4793	217	758	16	3639	360
Ba/F3-HLA-DQ2.2	10	1	2666	131	3065	151	2653	286	25795	585	15732	705	13453	191

Data are from an assay performed in triplicate (n=3). Those Ba/F3 cell lines are expressing HLA II with no peptide ligand as part of the construct.

Supplementary Table 5: Binding profile of antibodies against human B cells

Donor	Antibody	33mer gliadin peptide (-)		33mer gliadin peptide (+)		HLA-DQ allele 1	HLA-DQ allele 2
		MFI	%/DQN0139	MFI	%/DQN0139		
**953**	**Control Ab**	119	0.0%	106	0.0%	**DQ2.5**	DQ5.3
	**DONQ52**	128	0.5%	445	12.9%		
	**DQN0139**	1941	100.0%	2740	100.0%		
**1108**	**Control Ab**	124	0.0%	123	0.0%	**DQ2.5**	DQ6.3
	**DONQ52**	151	1.5%	348	10.2%		
	**DQN0139**	1886	100.0%	2331	100.0%		
**1139**	**Control Ab**	87	0.0%	85	0.0%	**DQ2.5**	DQ9.2
	**DONQ52**	115	1.4%	276	7.7%		
	**DQN0139**	2055	100.0%	2564	100.0%		
**1432**	**Control Ab**	109	0.0%	108	0.0%	**DQ2.5**	DQ8.1
	**DONQ52**	118	0.4%	316	9.1%		
	**DQN0139**	2191	100.0%	2391	100.0%		
**1436**	**Control Ab**	120	0.0%	119	0.0%	**DQ2.5**	DQ5.3
	**DONQ52**	129	0.9%	322	16.3%		
	**DQN0139**	1143	100.0%	1366	100.0%		
**1471**	**Control Ab**	109	0.0%	105	0.0%	**DQ2.5**	DQ6.3
	**DONQ52**	135	2.0%	291	12.3%		
	**DQN0139**	1410	100.0%	1623	100.0%		
**1516**	**Control Ab**	134	0.0%	130	0.0%	**DQ2.5**	
	**DONQ52**	163	1.4%	539	19.2%		
	**DQN0139**	2176	100.0%	2257	100.0%		
**1672**	**Control Ab**	121	0.0%	117	0.0%	**DQ2.5**	DQ6.2
	**DONQ52**	133	1.0%	250	11.4%		
	**DQN0139**	1312	100.0%	1287	100.0%		
**951**	**Control Ab**	94	0.0%	97	0.0%	DQ7.5	DQ6.4
	**DONQ52**	106	0.9%	98	0.1%		
	**DQN0139**	1447	100.0%	1365	100.0%		
**1015**	**Control Ab**	104	NC	101	NC	DQ5.1	DQ5.2
	**DONQ52**	109	NC	108	NC		
	**DQN0139**	113	NC	109	NC		
**1053**	**Control Ab**	139	0.0%	120	0.0%	DQ7.4	
	**DONQ52**	130	-0.3%	138	0.6%		
	**DQN0139**	3456	100.0%	3134	100.0%		
**1110**	**Control Ab**	107	NC	115	NC	DQ5.2	DQ6.3
	**DONQ52**	111	NC	111	NC		
	**DQN0139**	104	NC	107	NC		

Individual numerical data of DONQ52 binding to HLA-DQ2.5+ (n=8) human B cells or HLA-DQ2.5- (n=4) human B cells determined by flow cytometry and corresponding HLA-DQ alleles. Relative values of MFI (%) of antibodies (DONQ52, Control Ab: 10 μg/m) to DQN0139 (10 μg/mL) were shown as %/DQN0139. Data are from an assay performed once for each individual donor.

Supplementary Table 6: Source of HLA-DQ2.5:gluten epitope restricted TCR sequence, and median inhibitory concentrations (IC_{50}) against the pathogenic gluten epitopes

TCR clone	Epitope Restriction (Target epitope)	Source	Stimulated synthetic gluten peptide	Final peptide concentration	IC50 (ng/mL)
**387.9**	DQ2.5-glia-α1a	Oslo University Hospital*	33mer gliadin	250 nM	0.222
**D2**	DQ2.5-glia-α2	Petersen J et.al (3)	33mer gliadin	250 nM	0.320
**370.2.25**	DQ2.5-glia-α1b	Oslo University Hospital *	33mer gliadin	250 nM	1.48
**442D.A.2**	DQ2.5-glia-ω1	Oslo University Hospital *	ω1/2 gliadin	25 µM	1.76
**578.42**	DQ2.5-glia-ω2	Oslo University Hospital *	ω1/2 gliadin	250 nM	0.162
**820.270**	DQ2.5-glia-γ1	Oslo University Hospital *	γ1 gliadin	250 nM	3.61
**430.1.41**	DQ2.5-glia-γ2	Oslo University Hospital *	γ2 gliadin	250 nM	751
**430.1.146**	DQ2.5-glia-γ3	Oslo University Hospital *	γ3 gliadin	250 nM	2.11
**430.1.36**	DQ2.5-glia-γ4a	Oslo University Hospital *	γ4a gliadin	100 µM	3090
**430.1.94**	DQ2.5-glia-γ4d	Oslo University Hospital *	γ4d gliadin	25 µM	50.7
**1468.2**	DQ2.5-hor-3a	Dahal-Koirala S et.al (4)	B/C hordein	100 µM	27.9

List of sources of TCR sequence used in in vitro studies and the correspondence of TCR expressed in αβTCR-knockout Jurkat-NFAT-Luc2, stimulating peptides, and their final concentrations. Final peptide concentration was determined as non-saturated TCR activation-inducing concentrations as described in Supplementary Fig. 2. The IC_{50} value of DONQ52 was defined as the concentration at which there was a 50 % decrease in luciferase activity. IC_{50} values were determined by JMP15.0.0 (SAS Institute Inc), using inverse estimation in a non-linear regression model. *TCR sequences were provided by Oslo University Hospital under a material transfer agreement.

Supplementary Table 7: Crystallization conditions, data collection and refinement statistics

	**DQN0344AE02 - HLA-DQ2.5:DQ2.5-glia-α1a**	**DQN0344AE02 - HLA-DQ2.5:DQ2.5-glia-α2**	**DQN0385AE01 - HLA-DQ2.5:DQ2.5-glia-γ2**	**DQN0385AE02 - HLA-DQ2.5:DQ2.5-hor-3a**
**PDB ID**	**8W83**	**8W84**	**8W85**	**8W86**
**Crystallization conditions**	0.1 M HEPES (pH7.5), 25.0% (w/v) Polyethylene glycol 1,000, and 20% (v/v) Glycerol as a cryoprotectant	80 mM di-Sodium malonate (pH 4.0), 9.6% (w/v) Polyethylene glycol 3,350, and 25% (v/v) Ethylene glycol as a cryoprotectant	0.1 M Ammonium sulfate, 10.0% (w/v) Polyethylene glycol 3,350, and 30% (v/v) Ethylene glycol as a cryoprotectant	0.1 M MES monohydrate (pH 5.5), 12.0% (w/v) Polyethylene glycol 8,000, 0.1 M Calcium acetate hydrate, and 30% (v/v) Ethylene glycol as a cryoprotectant
**Data collection**				
X-ray source	Photon Factory / BL17A	SPring-8 / BL45XU with the ZOO system (6)	Photon Factory / BL1A	SPring-8 / BL45XU with the ZOO system
Detector	PILATUS3 S 6M (Dectris)	PILATUS 6M (Dectris)	EIGER X 4M (Dectris)	PILATUS 6M (Dectris)
Wavelength (Å)	0.98000	1.00000	1.10000	1.00000
Space group	$P1\ 2_1\ 1$	$I2_1\ 2_1\ 2_1$	$C2\ 2\ 2$	$P1\ 2_1\ 1$
Cell dimensions *a, b, c* (Å)	87.49, 174.24, 129.66	82.86, 138.71, 201.93	150.53, 243.91, 136.08	73.70, 127.19, 130.37
Cell dimensions *α, β, γ* (°)	90.00, 93.17, 90.00	90.00, 90.00, 90.00	90.00, 90.00, 90.00	90.00, 104.45, 90.00
Resolution (Å)	129.5 - 2.8 (3.3 - 2.8)*	114.3 - 2.1 (2.3 - 2.1)*	136.1 - 2.8 (3.1 - 2.8)*	126.2 - 2.2 (2.5 - 2.2)*
*R*merge	0.500 (1.237)*	0.078 (1.421)*	0.120 (1.883)*	0.102 (1.229)*
*R*meas	0.547 (1.358)*	0.083 (1.516)*	0.124 (1.956)*	0.113 (1.344)*
*R*pim	0.217 (0.551)*	0.028 (0.523)*	0.034 (0.524)*	0.048 (0.538)*
CC (1/2)	0.900 (0.593)*	0.999 (0.552)*	0.999 (0.684)*	0.998 (0.511)*
mean *I*/σ (*I*)	4.7 (1.7)*	17.1 (1.5)*	16.3 (1.6)*	10.7 (1.6)*
Total observations	285587 (13128)*	354515 (16425)*	571351 (28797)*	412512 (23238)*
Unique observations	44543 (2228)*	39315 (1966)*	41787 (2089)*	77604 (3880)*
Completeness (spherical) (%)	47.7 (6.7)*	58.1 (13.2)*	65.3 (11.3)*	69.1 (14.0)*
Completeness (ellipsoidal) (%)	92.7 (72.8)*	93.4 (61.4)*	94.0 (64.8)*	93.6 (61.4)*
Redundancy	6.4 (5.9)*	9.0 (8.4)*	13.7 (13.8)*	5.3 (6.0)*
Refinement				
Resolution (Å)	54.9 - 2.8 (3.1-2.8)*	114.3 - 2.1 (2.2-2.1)*	50.5 - 2.8 (3.0-2.8)*	126.2 – 2.2 (2.4-2.2)*
No. of reflections	44529	39315	41778	77604
*R*work /*R*free (%)†	27.79 / 32.09 (35.51 / 43.33)*	26.03 / 32.51 (30.84 / 38.24)*	25.30 / 29.99 (33.55 / 34.70)*	24.10 / 28.04 (30.85 / 34.72)*
Number of atoms	23643	6415	10295	11188
Number of chains	20	7	10	11
Number of amino acid residues	3145	812	1376	1395
Number of water molecules	0	167	0	352
Average B values	39.44	52.44	85.53	54.72
r.m.s.d. Bond lengths (Å)	0.004	0.008	0.008	0.008
r.m.s.d. Bond angles (°)	0.59	1.01	1.05	1.03
Ramachandran analysis‡ Outliers,Allowed,Favored (%)	0.81, 6.17, 93.02	0.38, 5.39, 94.24	1.12, 8.49, 90.39	0.37, 4.10, 95.54
Rotamer Outliers (%)‡	5.80	7.41	8.77	4.23
Clash Score ‡	4.35	6.59	7.43	4.45

The paratope residues recognizing peptides are conserved between DQN0344AE02 and DONQ52, DQN0385AE01 and DONQ52. The paratope residues recognizing peptides except for Glu28 are conserved between DQN0385AE02 and DONQ52. * Values in parentheses are for the highest-resolution shell. † Rfree is calculated with 5% of the reflection randomly set aside. ‡ Calculated with the program MolProbity (5).

Supplementary Table 8: List of antibodies used in flow cytometry experiments with experimental dilutions

Antibody	Catalogue number	Clone	Company	Dilution used
Anti human IgG-PE	2043-09	Polyclonal	Southern Biotech	1:50
Anti mouse IgG2a-PE	1082-09	Polyclonal	Southern Biotech	1:50
Anti human CD3-PE-Cy7	557749	SP34-2	BD Biosciences	1:100
Anti human CD14-Pacific Blue	558121	M5E2	BD Biosciences	1:100
Anti human CD16-FITC	555406	3G8	BD Biosciences	3:100
Anti HLA-DR-PerCP	347364	L243	BD Biosciences	3:100

Supplementary Table 9: List of gRNA sequences used generate endogenous αβTCR-knockout Jurkat-NFAT-Luc2

Name	Target DNA sequence	PAM
TRBC-1	GCAAACACAGCGACCTCGGGT	GGG
TRBC-2	GTGGCTCAAACACAGCGACCT	CGG
TRBC-3	GAGCTCAGCTCCACGTGGTCG	GGG
TRBC-4	GCGGCTGCTCAGGCAGTATC	TGG
TRAC-1	GCTGGTACACGGCAGGGTCA	GGG
TRAC-2	GAGAATCAAAATCGGTGAAT	AGG
TRAC-3	GATTAAACCCGGCCACTTTC	AGG
TRAC-4	GACAAAACTGTGCTAGACATG	AGG

Supplementary Table 10: PK parameters of the anti-HLA DQ antibodies in DQ2.5 Tgm

		*PK parameters									
**Group: Dose, Route**	**Feed**	**Cmax (µg/mL)**	**Tmax (d)**	**C0 (µg/mL)**	**T1/2 (d)**	**AUCinf (µg·d/mL)**	**CL (mL/d/kg)**	**CL/F (mL/d/kg)**	**Vss (mL/kg)**	**Vz/F (mL/kg)**	**F (%)**
**DONQ52: 0.1 mg/kg, IV**	GFD	-	-	2.64	1.71	2.52	39.8	-	71.3	-	-
**DONQ52: 0.5 mg/kg, IV**	GFD	-	-	13.3	2.76	NA†	NA†	-	NA†	-	-
**DONQ52: 2 mg/kg, IV**	GFD	-	-	57.4	4.09	149	13.5	-	72.7	-	-
**DONQ52: 10 mg/kg, IV**	GFD	-	-	288	4.85	1070	9.38	-	73.7	-	-
**DONQ52: 2 mg/kg, SC**	GFD	15.4	3.00	-	2.66	120	-	16.6	-	63.8	80.5
**DONQ52: 2 mg/kg, IV**	GCD	-	-	57.6	3.10	129	15.5	-	70.1	-	-
**DONQ52: 2 mg/kg, SC**	GCD	16.6	3.00	-	NC‡	NC‡	-	NC‡	-	NC‡	NC‡
**DQN0139: 0.1 mg/kg, IV**	GFD	-	-	2.50	0.0524	0.0702	1420	-	49.0	-	-
**DQN0139: 0.5 mg/kg, IV**	GFD	-	-	13.1	0.0872	1.83	273	-	41.3	-	-
**DQN0139: 2 mg/kg, IV**	GFD	-	-	54.8	0.276	26.1	26.1	76.5	-	34.1	-
**DQN0139: 10 mg/kg, IV**	GFD	-	-	294	1.62	332	NA§	NA§	-	NA§	-

Extended pharmacokinetic parameters of DONQ52 and DQN0139 in DQ2.5 Tgm. *Calculated parameters are Cmax (maximum drug concentration), Tmax (time to reach maximum concentration), C0 (Concentration at time 0), T1/2 (terminal phase half-life), AUCinf (area under the curve to infinity), CL (clearance), CL/F (subcutaneous clearance), Vss (steady-state volume of distribution), Vz/F (apparent volume of distribution in the terminal elimination phase) and F (bioavailability). F was calculated by the following formula: F (%) = (AUCinf / dose) at subcutaneous dosing / (AUCinf / dose) at intravenous dosing × 100. NA†: Not applicable, because the AUC%ext (43%) of AUCinf (=24.6 µg·d/mL) at 0.5 mg/kg exceeded 20%. The CL, Vss, calculated based on the reference value of AUCinf (24.6 µg·d/mL) at 0.5 mg/kg were 20.3 mL/d/kg, 74.3 mL/kg, respectively. NC‡ : Not calculated. NA§: Not applicable, because the AUC%ext (27%) of AUCinf (=455 µg·d/mL) at 10 mg/kg exceeded 20%. The CL, Vss, calculated based on the reference value of AUCinf (455 µg·d/mL) at 10 mg/kg were 22.0 mL/d/kg, 49.7 mL/kg, respectively. -: No data.

Supplementary Table 11: Grouping Details of in vivo study

Group	Dose (mg/kg)	Schedule	Route	n
**Non-treated**	none	none	none	10
**33mer gliadin peptide immunization with Control Ab treatment**	3 mg/kg loading, 1 mg/kg maintenance	Dosing day 0, 2, 4, 7	Intraperitoneal injection	10
**33mer gliadin peptide immunization with DQN0139 treatment**	Day 0, 4: 5mg/kg Day 2, 7: 4mg/kg	Dosing day 0, 2, 4, 7	Intraperitoneal injection	10
**33mer gliadin peptide immunization with DONQ52 treatment**	3 mg/kg loading, 1 mg/kg maintenance	Dosing day 0, 2, 4, 7	Intraperitoneal injection	10

Group	Dose (mg/kg)	Schedule	Route	n
**Non-treated**	none	none	none	10
**KLH immunization with vehicle treatment**	none	Dosing day 0, 2, 4, 7	Intraperitoneal injection	10
**KLH immunization with DQN0139 treatment**	Day 0, 4: 5mg/kg Day 2, 7: 4mg/kg	Dosing day 0, 2, 4, 7	Intraperitoneal injection	10
**KLH immunization with DONQ52 treatment**	3 mg/kg loading,1 mg/kg maintenance	Dosing day 0, 2, 4, 7	Intraperitoneal injection	10

Group	Dose (mg/kg)	Schedule	Route	n
**Non-treated**	none	none	none	10
**DQ2.5-glia-γ2 peptide immunization with Control Ab treatment**	13.23 mg/mL solution in the osmotic pump	Osmotic pump implantation day 0	Osmotic pump	10
**DQ2.5-glia-γ2 peptide immunization with DONQ52 treatment**	4.50 mg/mL solution in the osmotic pump	Osmotic pump implantation day 0	Osmotic pump	10

Grouping for the in vivo study to evaluate in vivo neutralizing activity of DONQ52 to the 33mer gliadin peptide (upper table), KLH (middle table) and DQ2.5-glia-γ2 epitope (lower table).

Supplementary References

1. J. A. Tye-Din et al., Comprehensive, quantitative mapping of T cell epitopes in gluten in celiac disease. Sci. Transl. Med. **2**, 41ra51 (2010).
2. L. M. Sollid, S. W. Qiao, R. P. Anderson, C. Gianfrani, F. Koning, Update 2020: nomenclature and listing of celiac disease–relevant gluten epitopes recognized by $CD4^+$ T cells. Immunogenetics **72**, 85-88 (2020).
3. J. Petersen et al., T-cell receptor recognition of HLA-DQ2-gliadin complexes associated with celiac disease. Nat. Struct. Mol. Biol. **21**, 480–488 (2014).
4. S. Dahal-Koirala et al., Discriminative T-cell receptor recognition of highly homologous HLA-DQ2-bound gluten epitopes. J. Biol. Chem. **294**, 941-952 (2019).
5. I. W. Davis et al., MolProbity: All-atom contacts and structure validation for proteins and nucleic acids. Nucleic Acids Res. **35**, W375–W383 (2007).
6. K. Hirata et al., ZOO: An automatic data-collection system for high-throughput structure analysis in protein microcrystallography. Acta Crystallogr. D Struct. Biol. **75**, 138–150 (2019).
