# Supplementary material for: Characterizations of a neutralizing antibody broadly reactive to multiple gluten peptide:HLA-DQ2.5 complexes in the context of celiac disease
Source: Nat Commun. 2023 Dec 22;14:8502. doi: 10.1038/s41467-023-44083-4 (PMC10746718; doi:10.1038/s41467-023-44083-4)
Supplement: Supplementary file 3 — Reporting Summary [file 41467_2023_44083_MOESM3_ESM.pdf]

nature portfolio

Corresponding author(s): Dr. Tomoyuki Igawa

Last updated by author(s): Nov 15, 2023

Reporting Summary

Nature Portfolio wishes to improve the reproducibility of the work that we publish. This form provides structure for consistency and transparency in reporting. For further information on Nature Portfolio policies, see our Editorial Policies and the Editorial Policy Checklist.

Statistics

For all statistical analyses, confirm that the following items are present in the figure legend, table legend, main text, or Methods section.

n/a	Confirmed	
☐	☒	The exact sample size (n) for each experimental group/condition, given as a discrete number and unit of measurement
☐	☒	A statement on whether measurements were taken from distinct samples or whether the same sample was measured repeatedly
☐	☒	The statistical test(s) used AND whether they are one- or two-sided<br>*Only common tests should be described solely by name; describe more complex techniques in the Methods section.*
☒	☐	A description of all covariates tested
☒	☐	A description of any assumptions or corrections, such as tests of normality and adjustment for multiple comparisons
☐	☒	A full description of the statistical parameters including central tendency (e.g. means) or other basic estimates (e.g. regression coefficient) AND variation (e.g. standard deviation) or associated estimates of uncertainty (e.g. confidence intervals)
☐	☒	For null hypothesis testing, the test statistic (e.g. F, t, r) with confidence intervals, effect sizes, degrees of freedom and P value noted<br>*Give P values as exact values whenever suitable.*
☒	☐	For Bayesian analysis, information on the choice of priors and Markov chain Monte Carlo settings
☒	☐	For hierarchical and complex designs, identification of the appropriate level for tests and full reporting of outcomes
☒	☐	Estimates of effect sizes (e.g. Cohen's d, Pearson's r), indicating how they were calculated

Our web collection on statistics for biologists contains articles on many of the points above.

Software and code

Policy information about availability of computer code

Data collection	BD FACSDiva Ver.8.0.1 software (Becton, Dickinson and Company), Biacore T200 (GE healthcare), EnVision (PerkinElmer), SoftMax Pro 6.4 (Molecular Devices), ImmunoSpot® S6 ENTRY (Software version: ImmunoSpot 5.1.36), Beamline BL17A / Beamline BL1A (Photon Factory), Beamline BL45XU (SPring8), PILATUS3 S 6M (Dectris), PILATUS 6M (Dectris), EIGER X 4M (Dectris)
Data analysis	JMP15.0.0 (SAS Institute Inc), Biacore T200 Evaluation Software Version 2.0 (GE Healthcare), Phoenix WinNonlin (version 6.4, Certara LP), Microsoft Excel 2016, JMP® (version 11.2.1 and version 15.0.0, JMP Statistical Discovery LLC), Prism 7.0 software (GraphPad Software, Inc.), autoPROC (Global Phasing), Phaser (developed by Randy Read's group at the Cambridge Institute for Medical Research (CIMR) in the University of Cambridge), Coot (Acta Crystallogr. D Biol. Crystallogr. 66, 486–501 (2010).) Buster (Global Phasing)

For manuscripts utilizing custom algorithms or software that are central to the research but not yet described in published literature, software must be made available to editors and reviewers. We strongly encourage code deposition in a community repository (e.g. GitHub). See the Nature Portfolio guidelines for submitting code & software for further information.

Data

Policy information about availability of data

All manuscripts must include a data availability statement. This statement should provide the following information, where applicable:

- Accession codes, unique identifiers, or web links for publicly available datasets
- A description of any restrictions on data availability
- For clinical datasets or third party data, please ensure that the statement adheres to our policy

The structure data generated in this study have been deposited in the Protein Data Bank (PDB) under the accession code 8W83 [https://doi.org/10.2210/pdb8W83/pdb], 8W84 [https://doi.org/10.2210/pdb8W84/pd], 8W85 [https://doi.org/10.2210/pdb8W85/pdb], and 8W86 [https://doi.org/10.2210/pdb8W86/pdb]. The structural data used in this study are available in the Protein Data Bank (PDB) under accession code 5KSA [https://doi.org/10.2210/pdb5ksa/pdb], 1S9V [https://doi.org/10.2210/pdb1s9v/pdb], 6XP6 [https://doi.org/10.2210/pdb6xp6/pdb], 4OZH [https://doi.org/10.2210/pdb4ozh/pdb], 4OZF [https://doi.org/10.2210/pdb4ozf/pdb], 4OZG [https://doi.org/10.2210/pdb4ozg/pdb]. The flowcytometry data generated in this study have been deposited in the FlowRepository database (https://flowrepository.org/) under accession code FR-FCM-xxx. The other source data generated in this study are provided in the Supplementary Information and the Source Data file. Other materials, including mAb sequence and protein information, can be provided to academic researchers for free via a material transfer agreement (MTA) that includes a research plan.

Research involving human participants, their data, or biological material

Policy information about studies with human participants or human data. See also policy information about sex, gender (identity/presentation), and sexual orientation and race, ethnicity and racism.

Reporting on sex and gender	This information has not been collected.<br>We used human PBMC derived CD4+T-cell to generate CD4+ T cell expressing TCRs specific for DQ2.5-glia-a2 for in vitro experiment. In this experiment, sex, gender, age have been anonymized, since sex, gender are considered not affecting the outcome of the experiment.
Reporting on race, ethnicity, or other socially relevant groupings	Japanese
Population characteristics	This information has not been collected. Reason: see avobe "Reporting on sex and gender"
Recruitment	Our study participants were recruited randomly from the employees of Chugai Pharmaceutical Co., Ltd. We did not set any specific selection criteria for this study. Prior to the blood draw, each participant was required to provide informed consent. This process involved a detailed discussion of the study, potential risks and benefits, and participant rights. Only after obtaining informed consent did we proceed with the blood draw.
Ethics oversight	Chugai Ethical Committee at Chugai Pharmaceutical Co., Ltd.

Note that full information on the approval of the study protocol must also be provided in the manuscript.

Field-specific reporting

Please select the one below that is the best fit for your research. If you are not sure, read the appropriate sections before making your selection.

☒ Life sciences ☐ Behavioural & social sciences ☐ Ecological, evolutionary & environmental sciences

For a reference copy of the document with all sections, see nature.com/documents/nr-reporting-summary-flat.pdf

Life sciences study design

All studies must disclose on these points even when the disclosure is negative.

Sample size	Sample sizes for experiments (other than structural analysis related experiments) were determined based on the reproducibility or stability of our preliminary experimental outcomes. During the preliminary stage of our research, we conducted a series of initial experiments to assess the feasibility of our study design and to observe the variability . These preliminary experiments provided us valuable insights into the expected variability. We also took into account practical considerations, such as the availability of resources and the time required to conduct the experiments, when determining the sample sizes. Therefore our sample sizes are sufficient to provide reliable and meaningful results, although we did not conduct sample size calculation<br>For structure analysis, because results of structure analysis are often unambiguous and considering availability of resources and the time required, we decided to determine sample size=1
Data exclusions	In Mice PK, 2mg/kg IV, GFD group, individual data from mice: 72hr (n=2/4), 168hr(n=3/4), 240hr(n=1/4) were excluded. 2mg/kg SC, GFD group, individual data from mice: 72hr (n=3/4), 168hr(n=2/4), 240hr(n=3/4) were excluded. 2mg/kg SC, GCD group, individual data from mice: 72hr (n=3/4), 168hr(n=3/4), 240hr(n=3/4) were excluded. We excluded those individual data because those animals developed anti-drug antibodies (ADA).
Replication	Replication of experiments were conducted except for structure analysis, because results of structure analysis are unambiguous.<br>All other experiments were replicated at least 2 times. The first experiment was conducted as a test that also serves to examine the

conditions, and the final experimant was carried out as the main experiment.

Randomization	Mice were randomized into different groups before experiments. The mice were randomized based on body weight by JMP® (version 11.2.1, JMP Statistical Discovery LLC.<br>For experiments other than those involving mice, randomization is considered not relevant, because these studies are conducted in a highly controlled environment, and involve cells or molecules rather than whole organisms. In these studies, the variables are limited.
Blinding	No blinding throughout the experiments. Since all of our experiments are nonclinical studies which has been conducted in a laboratory setting, and outcomes are coming from objective measurements, we considered blinding is considered not relevant for our study.

Reporting for specific materials, systems and methods

We require information from authors about some types of materials, experimental systems and methods used in many studies. Here, indicate whether each material, system or method listed is relevant to your study. If you are not sure if a list item applies to your research, read the appropriate section before selecting a response.

Materials & experimental systems

n/a	Involved in the study	
☐	☒	Antibodies
☐	☒	Eukaryotic cell lines
☒	☐	Palaeontology and archaeology
☐	☒	Animals and other organisms
☒	☐	Clinical data
☒	☐	Dual use research of concern
☒	☐	Plants

Methods

n/a	Involved in the study	
☒	☐	ChIP-seq
☐	☒	Flow cytometry
☒	☐	MRI-based neuroimaging

Antibodies

Antibodies used	Anti-gliadin antibody (clone 14D5, Abcam, ab36729), Goat F(ab')2 anti-Human IgG, Mouse ads-PE (Southern Biotech, 2043-09), SPV-L3 (anti-pan HLA-DQ antibody, Beckman Coulter, IM0416), Tu39 (anti-HLA-DR, DP, DQ antibody, BioLegend, 361702), MOPC-173 (mouse IgG2a isotype antibody, BioLegend, 400202), Goat F(ab')2 anti-Mouse IgG2a, Human ads-PE (Southern Biotech, 1082-09), PE-Cy7 Mouse Anti-Human CD3 (Clone SP34-2, BD Biosciences, 557749), Pacific Blue Mouse Anti-Human CD14 (Clone M5E2, BD Bioscience, 558121), FITC Mouse Anti-Human CD16 (Clone 3G8, BD Bioscience, 555406), Anti-human CD20 PE (Clone 2H7, BD Bioscience, 556633), PerCP anti-human HLA-DR Antibody (Clone L243, BD Bioscience, 347364), anti-human MHC Class I antibody (Clone: W6/32, Bio X Cell, Inc, BE0079), anti-HLA-DR antibody (Clone: L243, Bio X Cell, Inc, BE0306), DONQ52, DQN0139, Control antibody (IC17), and anti-human IgG-Fc monoclonal antibody (clone YG55) were purified in house.
Validation	All commercial available antibodies used were validated or published in numerous studied (for example, PMID: 29473905, PMID: 18252695, PMID: 2474601, PMID: 31185212, PMID: 11042292, PMID: 29170670, PMID: 23279566, PMID: 26719533).<br>For anti-human IgG-Fc monoclonal antibody (Southern Biotech, 2043-09), maker website states for species cross-reactivity: Human IgM and IgA; mouse immunoglobulins and pooled sera; may react with IgG from other species, and validated in various paper (PMID: 18252695, PMID: 37771579, PMID: 27356950).<br><br>Purity of in-house purified antibodies (DONQ52, DQN0139, IC17, and anti-human IgG-Fc monoclonal antibody (clone YG55)) was confirmed via SAS-PAGE (>95%). With regard to validation for the species, DONQ52 does not binding to B-cells from cynomolgus monkey nor mouse-pro B cell (Ba/F3 cell line) by FACS experiment, whereas DQN0139 binds to cynomolgus monkey B-cells but not bind to mouse-pro B cell (Ba/F3 cell line). IC17 is published in PMID: 35293276. anti-human IgG-Fc monoclonal antibody (clone YG55) is validated in patent (WO2019/112027A1), filed by Chugai Seiyaku Kabushiki Kaisha.

Eukaryotic cell lines

Policy information about cell lines and Sex and Gender in Research

Cell line source(s)	・Ba/F3 cells: Mouse pro-B cell line derived from C3H strain were sourced from Riken Cell bank, The sex of Ba/F3 cells is unknown<br>・IHW09023 cell lines: An Epstein-Barr virus (EBV) transformed lymphoblastoid cell line with HLA-DQ2.5. This cell line was sourced from The International Histocompatibility Working Group (IHW).The sex of IHW09023 cells is female
Authentication	HLA expression on various HLA overexpressed Ba/F3 cells were confirmed via anti-HLA antibodies. HLA-DQ2.5 expression in IHW09023 was confirmed in The International Histocompatibility Working Group/ Fred Hutchinson Cancer Center, and validated in housevia peptide dependent activation of gluten peptide:DQ2.5 restricted TCR expressed Jurkat NFAT-Luc cells.
Mycoplasma contamination	Confirmed all tested cell lines tested negative for mycoplasma contamination by PCR
Commonly misidentified lines (See ICLAC register)	No commonly misidentified cell lines were used in the study

Animals and other research organisms

Policy information about studies involving animals; ARRIVE guidelines recommended for reporting animal research, and Sex and Gender in Research

Laboratory animals	HLA-DQ2.5 transgenic mice (H2-Ab1 KO/HLA-DQ2.5 transgenic mouse), male, 6-8 weeks old.<br>NZW rabbits, female, 12-16 weeks old
Wild animals	No wild animals were used in the study
Reporting on sex	Male mice were used, because for continuous bleeding of transgenic mice.<br>Sex-based difference in antibody PK and immunological responses were not observed in preliminary experiments<br><br>Female rabbits were used, because female NZW rabbit has been used in many immunizations for antibody production in the past.
Field-collected samples	No field collected samples were used in the study
Ethics oversight	All animal studies were performed according ARRIVE (Animal Research: Reporting of In Vivo Experiments) guidelines (44). All procedures associated with this study were reviewed and approved by the Institutional Animal Care and Use Committee (IACUC) in Chugai Pharmaceutical Co., Ltd. The test facility is fully accredited by the Association for Assessment and Accreditation of Laboratory Animal Care International (AAALAC), a non-profit organization that promotes the humane treatment of animals in science through voluntary accreditation and assessment programs (http://www.aaalac.org). Animal care and experiments were performed according to the animal husbandry policy of Chugai Pharmaceutical Co., Ltd.

Note that full information on the approval of the study protocol must also be provided in the manuscript.

Plants

Seed stocks	No seed stocks were used in the study
Novel plant genotypes	No novel plant genotypes were used in the study
Authentication	Not applicable

Flow Cytometry

Plots

Confirm that:

- ☐ The axis labels state the marker and fluorochrome used (e.g. CD4-FITC).
- ☐ The axis scales are clearly visible. Include numbers along axes only for bottom left plot of group (a 'group' is an analysis of identical markers).
- ☐ All plots are contour plots with outliers or pseudocolor plots.
- ☐ A numerical value for number of cells or percentage (with statistics) is provided.

Methodology

Sample preparation	Primary antibodies were incubated with Ba/F3 cell panels in FACS buffer (DPBS(−) containing 2% heat inactivated FBS and 2 mM EDTA) for 30 minutes at room temperature. Cells were stained with secondary antibodies, then cells were washed out with FACS buffer and examined by flow cytometry.<br><br>PBMCs were cultured in the presence or absence of 33mer gliadin peptide for 3 hours in 5% CO_2 at 37 °C. PBMCs were re-distributed on a 96-well plate, followed by incubation with primary antibodies for 1 hour at 4 °C. Cells were stained with secondary antibodies. Cells were washed out with FACS buffer and examined by flow cytometry.
Instrument	BD LSRFortessa X-20, Becton, Dickinson and Company
Software	BD FACSDiva Ver.8.0.1 software (Becton, Dickinson and Company)
Cell population abundance	For each run, >10,000 cells were analyzed based on previous experiment.
Gating strategy	For binding study using Ba/F3, FSC/SSC gates were done to exclude debris.

For binding study using PBMC, FSC/SSC gates were done to exclude debris. The boundary between positive and negative staining cells for CD3, CD14, CD16, CD20 was determined based on clearly distinguished population. Finally we determined CD3-, CD14-, CD16-, CD20+ population as B-cell.

☒ Tick this box to confirm that a figure exemplifying the gating strategy is provided in the Supplementary Information.
